# Stereoselective Interactions of Chiral Polyurea Nanocapsules with Albumins

**DOI:** 10.1021/acsami.4c09565

**Published:** 2024-08-23

**Authors:** Amani Zoabi, Adan Sultan, Malak Abo Alhija, Sergei Remennik, Anna Radko, Katherine Margulis

**Affiliations:** † The Institute for Drug Research, the School of Pharmacy, the Faculty of Medicine, The Center for Nanoscience and Nanotechnology, The Hebrew University of Jerusalem, Jerusalem 9112192, Israel; ‡ The Unit for Nanoscopic Characterization, The Center for Nanoscience and Nanotechnology, The Hebrew University of Jerusalem, Jerusalem 91904, Israel

**Keywords:** albumin adsorption, chiral polymeric nanocapsules, morphology, stereoselective interactions, polyurea, cell internalization

## Abstract

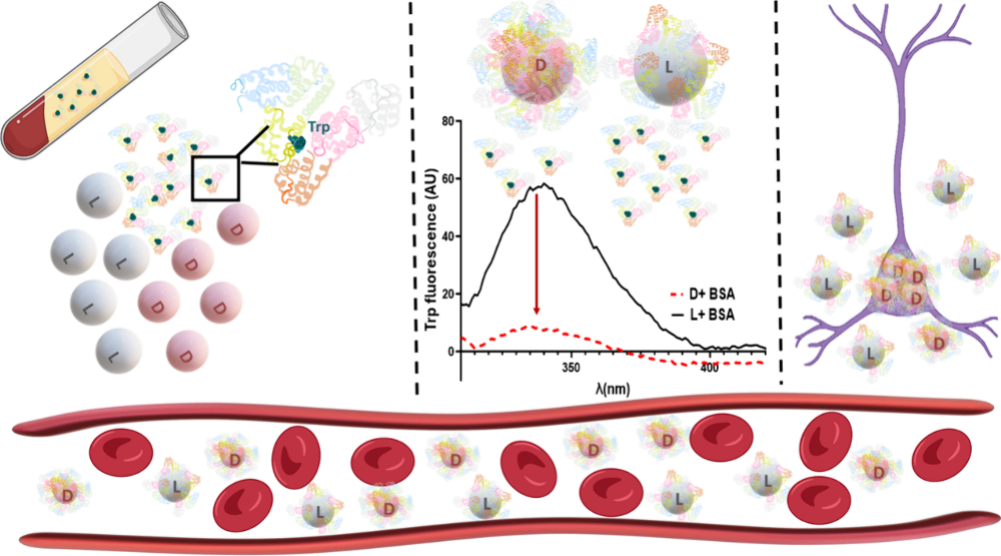

Exploiting the chirality of nanometric structures to modulate biological systems is an emerging and compelling area of research. In this study, we reveal that chiral polyurea nanocapsules exhibit significant stereoselective interactions with albumins from various sources despite their nearly neutral surface potential. Moreover, these interactions can be modulated by altering the nanocapsule surface composition, offering new opportunities to impact their distribution and, if used as a drug delivery system, the pharmacokinetics of the drug. Notably, these interactions promote preferential cellular internalization of only one chiral configuration. We synthesized chiral polyurea nanocapsules with reproducible sizes via interfacial polymerization between toluene 2,4-diisocyanate and d- or l-lysine enantiomers on a volatile oil-in-water emulsion interface, followed by solvent evaporation. Further synthesis optimization reduced the capsule size to a range compatible with *in vivo* administration, and capsules with alternating chiral patterns were also produced. The stereoselective interactions with albumins were assessed through capsule size changes, fluorescence quenching, and surface charge measurements. Biocompatibility, stability, and cellular internalization were evaluated. Additionally, scanning transmission electron and atomic force microscopy were carried out to assess the capsule shape, surface composition, and morphology. We discovered that d-nanocapsules exhibited 2.1–2.6 times greater albumin adsorption compared with their l-counterparts. This difference is attributed to the distinct morphology of d-nanocapsules, characterized by a more concave shape, central depression, and rougher surface. The extent of adsorption could be finely tuned by adjusting the d- and l-lysine monomer ratios during synthesis. Both chiral configurations demonstrated biocompatibility and stability with d-nanocapsules showing a 2.5-fold increase in cellular internalization.

## 1 Introduction

Chirality is prevalent in nature, and a majority of important biomolecules are chiral. Consequently, a biological activity often depends on stereoselective interactions, rendering many pharmaceuticals chiral.^1,2^ Despite the widespread use of individual chiral active molecules, exploiting the entire nanometric chiral structures as a platform for achieving specific *in vivo* interactions or for facilitating drug delivery, has only recently started being explored.^2−4^ Still, the majority of chiral nanostructures proposed for use in biomedical applications are not intrinsically chiral but rather feature nonchiral cores decorated with an external layer of individual chiral molecules.^2,5,6^ The exploration of intrinsically chiral nanostructures in biomedicine remains very limited. Yet, such inherently chiral structures are compelling not only because of their potential enantioselective behavior but also because of the possible impact of chirality on their physical attributes, including size, shape, and surface morphology.

In this study, we reveal that chiral polyurea nanocapsules synthesized by an interfacial polyaddition reaction in volatile emulsion exhibit stereoselective interactions with albumins from different sources. The impact of d- and l-configurations on the shape and surface morphology of these intrinsically chiral nanocapsules is being explored, and their possible roles in stereoselective interactions are discussed.

Albumins are the main plasma proteins.^7^ Interactions of drugs or their carriers with albumins determine the pharmacokinetics, pharmacodynamics, biodistribution, half-life, and clearance of the former.^8−10^ Because these metrics are of utmost importance in drug delivery, the albumin binding mechanisms are carefully investigated in the drug development process.^8,10^ The ability to predict and, to a certain extent, affect these interactions is imperative for the development of successful drug products. In addition to biodistribution and pharmacokinetics, interactions with albumin also affect cell permeation, because albumin coating induces cellular internalization.^8,11−14^

Albumin binding depends on many parameters, including hydrophobicity, charge, size, chemical structure, and hydrogen bonding formation abilities of associating molecules.^15^ While the influence of drug molecule chirality on albumin binding is widely acknowledged, likely due to stereospecific fitting into protein binding sites, the effect of the chirality of an entire nanometric structure on albumin binding is rarely documented, with a few exceptions.^16,17^

**Figure 1:**
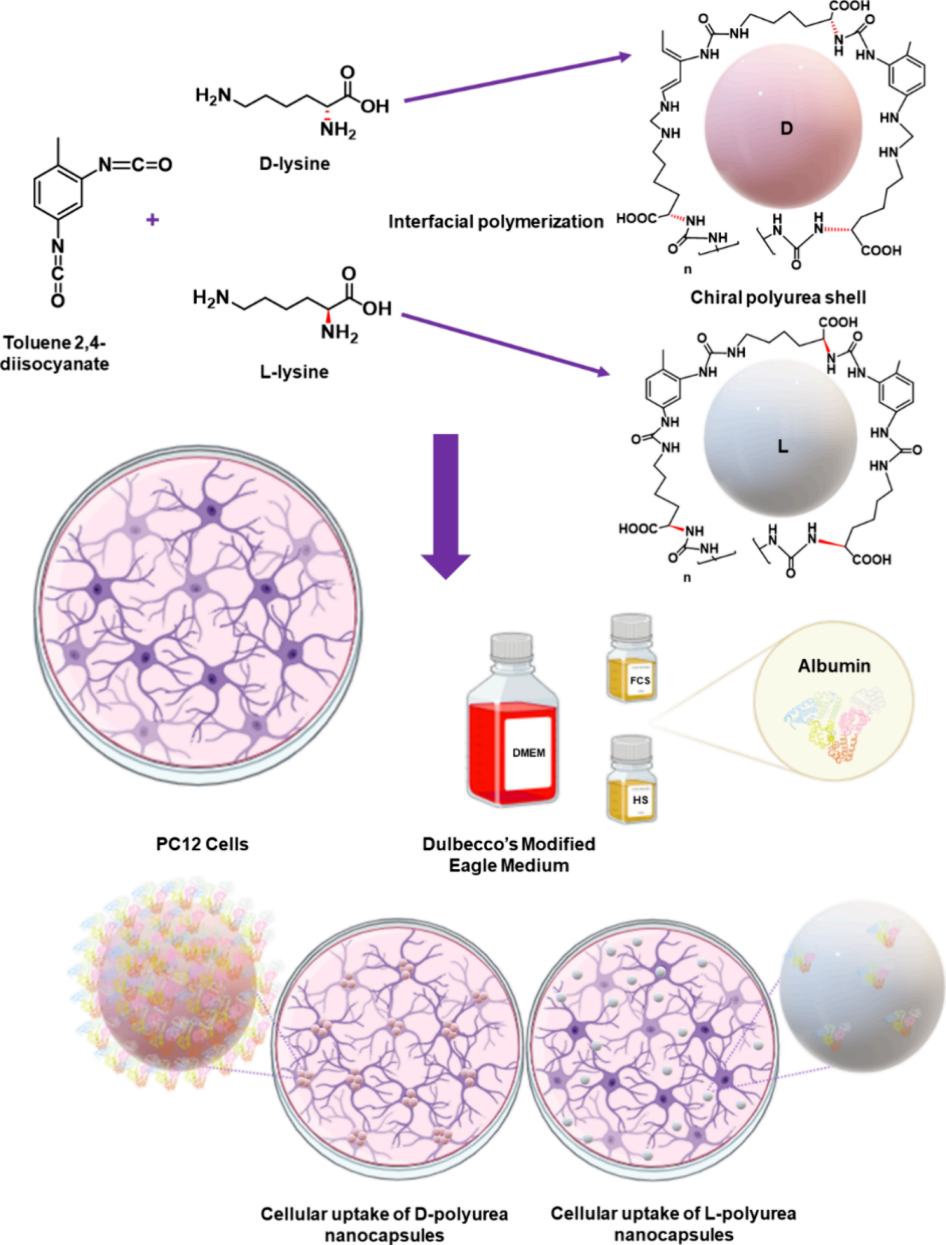
Schematic representation of this study. Chiral polyurea nanocapsules were synthesized by the interfacial polyaddition reaction between toluene 2,4-diisocyanate and lysine enantiomers. The stereoselective interactions of these shells with albumins from different sources are demonstrated by the preferential uptake of d-nanocapsules by PC12 cells from cell media containing a mixture of albumins.

Polyurea nanocapsules are biocompatible and biodegradable, and can facilitate the controlled delivery of a wide variety of drugs.^18−21^ They were demonstrated to protect drugs from premature degradation *in vivo* and enable targeted delivery.^20^ However, the synthesis of chiral polyurea nanocapsules is not common, with only a few published studies in the field.^22,23^ Herein, we report that optimization of the interfacial polyaddition reaction between toluene 2,4-diisocyanate and lysine enantiomers leads to the formation of chiral nanocapsules in the size range relevant to drug administration. These nanocapsules are biocompatible and stable in aqueous dispersions. We not only describe the stereoselective interactions of chiral polyurea nanocapsules with albumins but also show that these interactions promote stereoselective cellular internalization of the capsules (Figure 1).

We further demonstrate how the extent of interactions can be controlled by combining different ratios of chiral lysine monomers during the synthesis reaction.

## 2 Experimental Section

### 2.1 Materials

Toluene 2,4-diisocyanate (TDI), poly(vinyl alcohol) (PVA, average m.w. ≈ 67,000), poly(vinyl alcohol), 87–90% hydrolyzed (hydrolyzed PVA, average m.w. ≈ 30,000–70,000), *n*-butyl acetate, d-lysine monohydrochloride, l-lysine monohydrochloride, fluorescein isothiocyanate (FITC), thiazolyl blue tetrazolium (MTT reagent), bovine serum albumin, and human serum albumin were purchased from Sigma-Aldrich (St. Louis, MO, USA). Rat plasma was prepared by centrifuging the blood extracted from Sprague–Dawley (SD) rats (male and female, a strain of the Hebrew University, originally obtained from Harlan, Jerusalem, Israel). Double distilled water (DDW) was used for all experiments. Dulbecco’s modified Eagle’s medium (DMEM), fetal calf serum, horse serum, trypsin-EDTA 0.025%, Pen-Strep solution, and phosphate buffered saline (PBS) (1×, pH 7.4) were purchased from Sartorius (Beit Haemek, Israel). PC12 cell line was obtained from ATCC (Manassas, VA USA).

### 2.2 Methods

#### 2.2.1 Synthesis of Chiral Nanocapsules

Chiral polyurea nanocapsules were prepared by interfacial polyaddition reaction between toluene 2,4-diisocyanate and d- or l-lysine enantiomers. The interfacial polyaddition was initiated by emulsification of a volatile organic phase containing 4.75 g of *n*-butyl acetate and 0.25 g of toluene 2,4-diisocyanate (TDI) with 80 g of 2.5 wt % PVA solution in water (aqueous phase, preprepared at 80 °C with subsequent cooling to room temperature). The emulsification was carried out by ultrahigh sonication for 50 min using a VCX-750 ultrasonic liquid processor (Sonics and Materials, Inc., Newtown, CT, USA) with an output of 750 W at 20 kHz. This enabled the dispersion of the organic phase as nanosized droplets. Then, 0.365 g of either l- or d-lysine monohydrochloride were dissolved in 14.4 g of DDW, and the resultant solutions were added gradually to the emulsion described above, followed by heating to 35 °C for 20 h under 1500 rpm continuous stirring to evaporate the volatile solvent and to form the hollow chiral nanocapsules (unmodified nanocapsules).

The size of the capsules was critically reduced (modified nanocapsules) by increasing the reaction temperature from 35 to 62 °C with tight temperature control (±2 °C, ensured by using IKA RCT basic stirring plate equipped with contact temperature controller (IKA, Staufen, Germany)) and by modifying the stabilizing agent from PVA to hydrolyzed PVA. All resultant nanocapsules were washed four times with DDW by centrifugation at 15200 rpm using a SL 16R centrifuge (Thermo Fisher Scientific, Waltham, MA, USA) to remove PVA before albumin adsorption.

#### 2.2.2 Synthesis of Shells of Mixed Chirality

The chirality was controlled by the addition of l- and d-lysine monohydrochloride enantiomers in different ratios to the aqueous phase of the emulsion. The unmodified capsule synthesis protocol was used (see Section 2.2.1). The enantiomers were premixed in each evaluated ratio prior to addition to the emulsion.

#### 2.2.3 Albumin Adsorption onto the Chiral Nanocapsules

250 μL of 1 wt % solution of albumins (from bovine or human origin) in PBS or 250 μL of rat plasma were added to 250 μL of 1 mg D- or L-chiral nanocapsule dispersions in PBS. The mixture was stirred for 2 h at 50 rpm under ambient conditions. For the modified nanocapsules, the percentage of albumin solution was decreased to 0.1 wt % and followed the same procedure.

#### 2.2.4 Dynamic Light Scattering and ζ-Potential Measurements

The size and ζ-potential were measured using a Zetasizer Nano model ZEN3600 (Malvern Panalytical, Malvern, UK). All measurements were performed in triplicate. Three different batches of each dispersion were evaluated. The dispersions were diluted 20-fold with DDW for size measurements or with 10 mM sodium chloride solution for ζ-potential measurements.

#### 2.2.5 Fluorescence Measurements

Tryptophan residue fluorescence quenching induced by the interactions of albumins with the nanocapsules was measured with a Cary Eclipse fluorescence spectrophotometer (Varian, USA). The fluorescence emission spectra were recorded in the wavelength range of 310 to 450 nm upon excitation at 295 nm. Additionally, the fluorescence of FITC-labeled nanocapsules was measured in the wavelength range of 450 to 600 nm upon excitation at 498 nm.

#### 2.2.6 Free and Adsorbed BSA/HSA Concentration Determination

The free BSA/HSA concentration was determined by creating a calibration curve by using various concentrations of BSA/HSA in a PBS solution. The measurements were taken by using a fluorometer with excitation at 295 nm and recording wavelengths between 310 and 450 nm. To determine the adsorbed BSA/HSA concentration, the concentration of albumins after quenching was subtracted from the initial concentration.

#### 2.2.7 Circular Dichroism

Circular dichroism (CD) measurements were performed with a MOS-500 spectrometer (BioLogic, France). The measurements were performed over a wavelength range of 200–250 nm by using a 1 cm path length cuvette. The spectral resolution was 1 nm. The reported spectra are the results of three scans.

#### 2.2.8 Scanning Transmission Electron Microscopy analysis

High-resolution high-angle annular dark field (HAADF) scanning transmission electron microscopy (STEM) images and elemental mapping were obtained on FEI Themis G3. Ten μL of dispersions were diluted 20-fold with DDW, as above, and placed on copper grid support films, Formvar/carbon, 400 mesh, Cu (Ted Pella, Inc., Redding, CA, USA). The resultant specimens were scanned at 300 keV acceleration voltages. For capsule shape statistic calculations, the images were processed with a high pass filter to achieve a sharper image of the particle shape, and concave particles with a central depression effect were counted using ImageJ software in the field comprising 100 particles.^24^

#### 2.2.9 Atomic Force Microscopy (AFM) Analysis

The chiral d- and l-nanocapsule dilute dispersions (0.005 wt %) were added to silicon wafers (Item# 452, University Wafers, Inc. South Boston, USA) overnight. Dimension Icon-XR SPM Atomic Force Microscopy (AFM) system (Bruker, USA) was used to characterize the chiral capsule morphology. Images were acquired in tapping mode, under ambient conditions, using RTESP-300 probes (Bruker, USA) (spring constant 42 N/m, frequency 300 kHz).

#### 2.2.10 MTT Assay

The MTT assay was used to evaluate the viability of PC12 cells following exposure to D- and L- polyurea nanocapsules. The cells were maintained at 37 °C under 5% CO2 in the medium supplemented with 7% (v/v) fetal calf serum, 7% (v/v) horse serum, and 1% (v/v) Pen-Strep solution. The medium was changed every 48 h. When the cells reached confluency, they were detached using 0.025% (w/v) trypsin and transferred to new culture flasks. After sufficient growth for experimentation, the cells were applied to 96-well plates at a density of 5 × 10^4^ cells/well. Each well contained 100 μL of cell suspension, and the plates were incubated for 24 h at 37 °C under 5% CO_2_ to obtain a monolayer culture. After 24 h of incubation, the medium was removed from each well. Thereafter, 100 μL of 1 mg/mL of d- and l-polyurea nanocapsules dispersed in complete cells medium was added. Cell viability was tested over 48 h of incubation at 37 °C, under 5% CO_2_, and changing the medium every 24 h. On the experiment day, 10 μL of MTT reagent (5 mg/mL) was added and incubated for 3 h at 37 °C under 5% CO_2_. Afterward, 300 μL of DMSO was added to dissolve the purple crystal formazan. After 20 min, the absorbance was measured using a Tecan microplate reader (TECAN, SPECTRA Fluor PLUS, Salzburg, Austria) at a measurement wavelength of 540 nm and a reference wavelength of 650 nm.

#### 2.2.11 FACS Analysis of Cellular Internalization of Fluorescently Labeled Nanocapsules

To evaluate the possible changes in the cell internalization capacity of the modified nanocapsules as a result of protein adsorption from cell media, FITC-labeled nanocapsules of opposite chiralities were prepared by dissolving FITC in the organic phase (0.0015 w/v %) and synthesizing the modified nanocapsules as detailed above (see Section 2.2.1).

PC12 cells were grown in Dulbecco’s modified Eagle’s medium (DMEM) supplemented with 7% (v/v) fetal calf serum, 7% (v/v) horse serum, and 1% (v/v) Pen-Strep solution. The cells were plated 4 × 10^5^ cells-per-well in six-well plates, to which the cells were allowed to adhere overnight under normal culture conditions. The medium was removed and the cells were incubated with 1 mg/mL FITC-labeled d- or l-nanocapsules dispersed in a complete cell medium for 2 h. The medium was removed again and the cells were washed twice with PBS. To assess the cellular uptake of fluorescently labeled nanocapsules, flow cytometry studies were performed. The cells washed twice with PBS after the incubation with FITC-labeled modified nanocapsules were removed using a cell-scraper. The cells were collected into 1.5 mL Eppendorf tubes and then sedimented for 5 min at 1300 rpm at room temperature. The supernatant was removed and the cells were resuspended with 500 μL cold PBS (4 °C) supplemented with 1% (w/v) BSA, and analyzed by a CytoFLEX flow cytometer equipped with 488 excitation laser (Beckman Coulter, Inc., Indianapolis, IN, USA) with gating based on normalized fluorescence of untreated cells.

#### 2.2.12 Live Cell Imaging–Confocal Microscopy

To visualize the cellular uptake of fluorescently labeled nanocapsules, live cell imaging by confocal microscopy was performed. Cells were plated on an eight-well chamber removable microscope-sterilized glass slide (ibidi, NBT Ltd., Or Akiva, Israel). The cells were allowed to adhere overnight under normal culture conditions. The medium was removed and the cells were incubated (37 °C, humidified atmosphere containing 5% CO_2_) with 300 μg FITC-labeled d- or l-modified nanocapsules dispersed in a complete cell medium (300 μL) for 2 h, followed by washing twice with PBS. For negative control, untreated cells were used. Cell fluorescence analysis was performed using a Nikon AX-R confocal microscope (Nikon Instruments, Melville, NY USA). To compare the cellular uptake of FITC-labeled d- and l-polyurea nanocapsules, region of interest (ROI) analysis was performed on the confocal images using NIS-Elements AR software. Average intensities of FITC were obtained by integrating them over the entire area of each cell.

## 3 Results and Discussion

### 3.1 Interactions of Chiral Nanocapsules With Purified and Plasma Albumins

**Figure 2:**
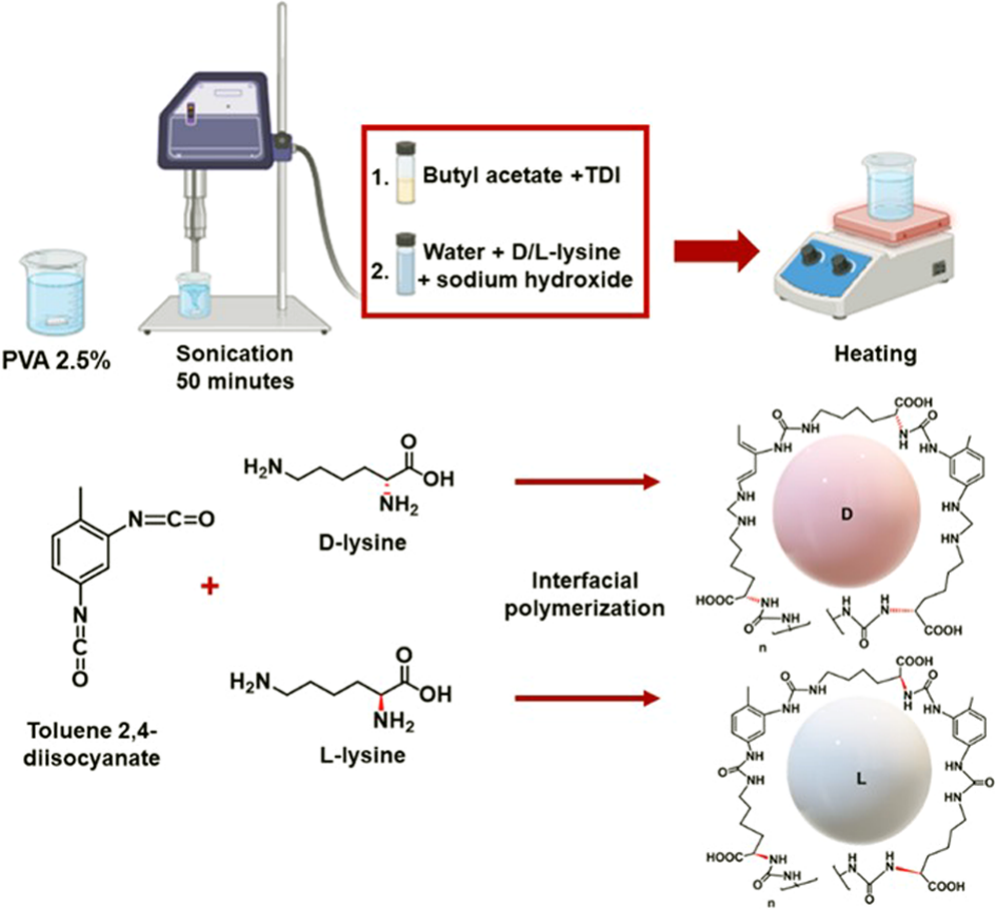
Representative scheme of the chiral polyurea nanocapsules preparation by interfacial polyaddition reaction between toluene 2,4-diisocyanate (TDI) and either d- or l-lysine enantiomers. The process starts with emulsifying a volatile organic phase (*n*-butyl acetate with dissolved TDI) with PVA aqueous solution by ultrahigh sonication, resulting in the dispersion of the organic phase as nanosized droplets. Then, the reaction is initiated on the droplet interface by adding a solution of either l- or d-lysine with sodium hydroxide gradually to the emulsion, followed by heating to 35 °C (or 65 °C for smaller “modified” capsules) under continuous stirring. This process results in the formation of chiral nanocapsules.

**Figure 3:**
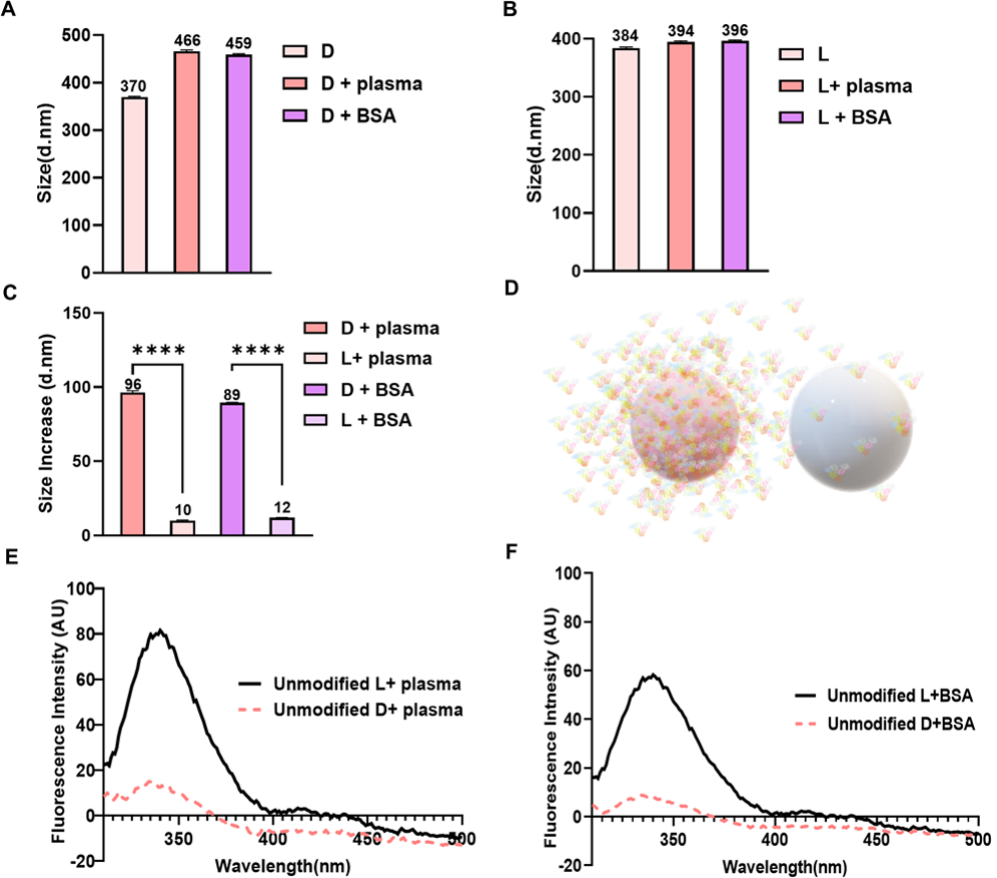
Interactions of chiral polyurea nanocapsules with albumins. (A) Representative size of unmodified d-polyurea nanocapsules before and after the adsorption of BSA and plasma. (B) Representative size of unmodified l-polyurea nanocapsules before and after adsorption of BSA and plasma. (C) Comparative analysis of the size increase after protein adsorption on d- and l-polyurea nanocapsules. Statistical analysis was performed by one-way analysis of variance with all pairwise multiple comparison procedures done by Tukey’s test. Symbols: ****P ≤ 0.0001. (D) Graphic presentation of protein adsorption on chiral capsules. (E) Fluorescence spectra after plasma equilibration with d-nanocapsules (dashed spectrum) and with l-nanocapsules (solid spectrum). (F) Fluorescence spectra after BSA equilibration with d-nanocapsules (dashed spectrum), and with l-nanocapsules (solid spectrum).

The chiral d- and l-capsules in the submicrometer size range (further referred to as “nanocapsules”) were formed by polymerization between toluene 2,4-diisocyanate and d- or l-lysine enantiomers on an interface of a volatile oil-in-water emulsion followed by volatile solvent evaporation (Figure 2). Lysine monomer chirality determined the overall chirality of the nanocapsules, as was confirmed by circular dichroism measurements and reported by us before.^23^ This synthesis yielded hollow shells with average sizes of 370 ± 2 nm for d-nanocapsules and 384 ± 2 nm for l-nanocapsules with polydispersity indexes of 0.13 ± 0.02 and 0.14 ± 0.01, respectively, as evaluated by dynamic light scattering (DLS) (Figure 3 and Figure S1). The shells had almost neutral ζ-potentials, 1.1 ± 1.3 mV for d-nanocapsules and −1.2 ± 1.5 mV for l-nanocapsules.

To evaluate the impact of capsule chirality on their interactions with albumin, bovine serum albumin (BSA) was introduced into aqueous dispersions of D- and L-nanocapsules at pH = 7.4,^25,26^ and the mixtures were equilibrated in ambient conditions.

To examine the adsorption of albumin to the capsule surface, capsule size increase and quenching of tryptophan residue fluorescence of albumins before and after the equilibration with nanocapsules were evaluated (Figure 3 and Figure S1). It can be clearly seen that markedly greater adsorption of albumin occurred on d-nanocapsules compared to their l-counterparts. This was evident from a more significant size increase after BSA adsorption (from 370 ± 2 nm to 459 ± 2 nm for d-nanocapsules and from 384 ± 2 nm to 396 ± 2 nm for l-nanocapsules) and a markedly more profound albumin fluorescence quenching.

Albumin is the major component of plasma and is responsible for carrying drugs in the bloodstream, their distribution, and pharmacokinetics. Therefore, to model the potential differences in the behavior of chiral nanocapsules when exposed to plasma components, the capsules were incubated with rat plasma. Figure 3 and Figure S1 demonstrate a differential size increase and albumin fluorescence quenching after the incubation with d- and l-nanocapsules with BSA and plasma. Again, a markedly greater size increase after incubation with rat plasma was detected for d-nanocapsules (from 370 ± 2 nm to 466 ± 3 nm) than for l-nanocapsules (384 ± 2 to 394 ± 2 nm). Also, the mixture fluorescence was quenched significantly stronger by d-nanocapsules, compared to their l-counterparts. These observations indicate preferential adsorption of rat plasma albumin (and potentially other proteins) onto d-nanocapsules.

### 3.2 Shells of Mixed Chirality

**Figure 4:**
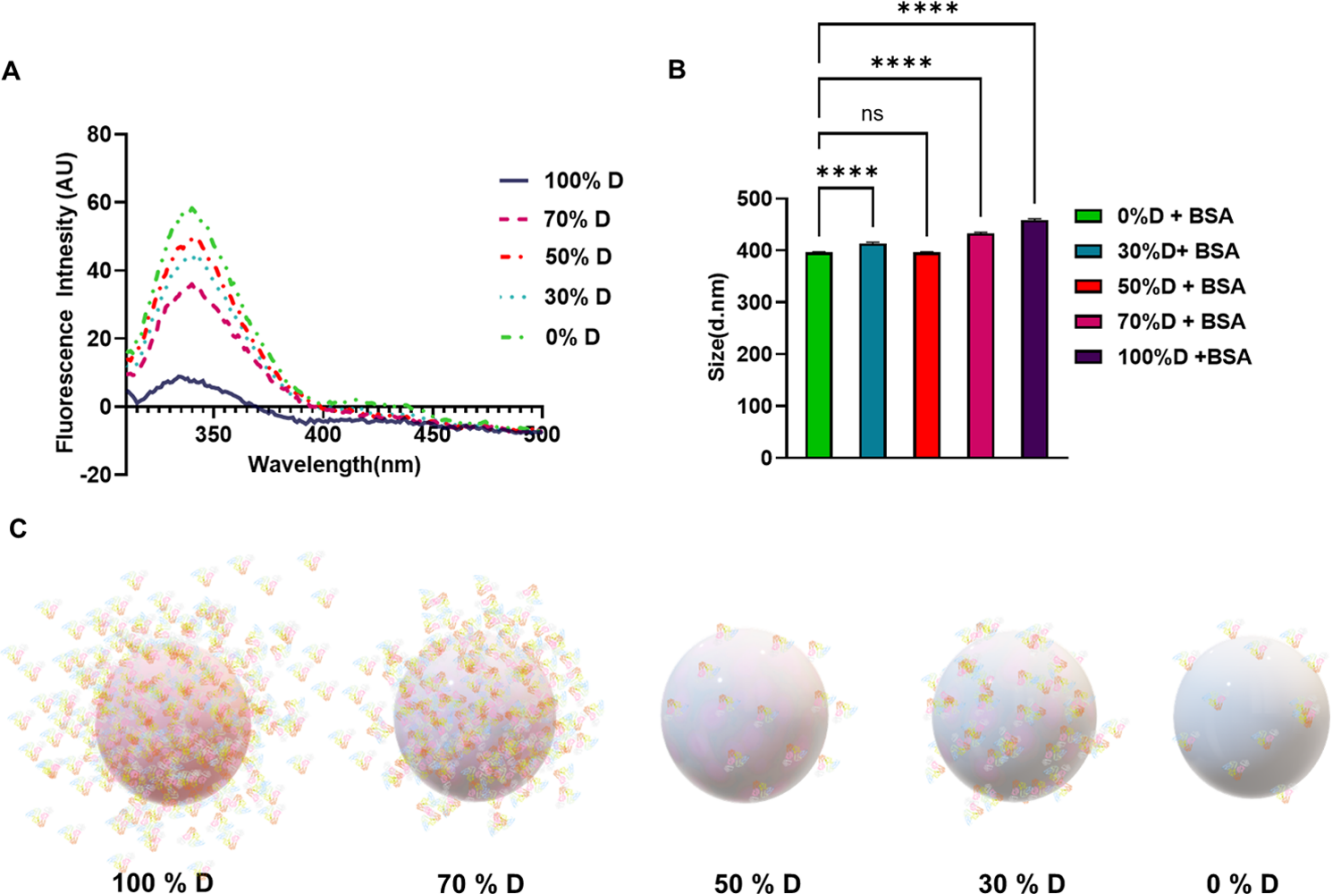
Albumin adsorption onto shells of mixed chirality. (A) Fluorescence spectra of BSA after adsorption on chiral nanocapsules formed by different ratios between d- and l-lysine enantiomers: 100% L in the shell, green spectrum; 30% D in the shell, blue spectrum; 50% D in the shell, orange spectrum; 70% D in the shell, pink spectrum; and 100% D in the shell, purple spectrum. (B) Representative size of chiral nanocapsules formed by different ratios between d- and l-lysine enantiomers after BSA adsorption. Statistical analysis was performed by one-way analysis of variance with all pairwise multiple comparison procedures done by Tukey’s test. Symbols: ns *P* ≥ 0.05, ****P ≤ 0.0001. (C) Graphic representation of capsules of mixed chirality formed with varying content of the d-monomer after albumin adsorption.

We next assumed that it would be possible to affect the extent of albumin adsorption onto the nanocapsules by introducing both enantiomeric monomers at different ratios during chiral shell synthesis. The idea was to create regions of varying chirality on the capsule surface to gain control over adsorption. Five different ratios between d-lysine and l-lysine monomers were investigated: 100% d-lysine, 70% d-lysine combined with 30% l-lysine, 50% d-lysine and 50% l-lysine, 30% d-lysine and 70% l-lysine, and finally 100% l-lysine. BSA was used in these experiments as a model albumin for the adsorption evaluation. The sizes of synthesized nanocapsules before BSA adsorption were 370 ± 2, 365 ± 2, 362 ± 2, 376 ± 2, and 384 ± 2 nm for 100:0, 70:30, 50:50, 30:70, and 0:100 (d:l) respectively. After equilibration with BSA for 2 h in ambient conditions, DLS measurements were performed and revealed the average size of 531 ± 1, 433 ± 2, 396 ± 2, 414 ± 2, and 387 ± 2 nm for 100:0, 70:30, 50:50, 30:70, and 0:100 (d:l), respectively, showing a general increase in diameter by increasing the percentage of d-lysine monomer in the shell, with the exception of 50:50 ratio (Figure 4, Figure S2).

It is evident from these results that the interactions become more pronounced with the rise in the content of the D monomer during the synthesis reaction, except for the 50:50 ratio, which probably results in racemic capsules. We assume that combining various ratios of lysine enantiomers leads to distinct regions of opposite chiralities on the capsule surface, creating a sort of mosaic pattern. Thus, the size and density of polymeric regions of opposite chiralities determine the extent of interactions with albumins. This uncovered phenomenon may allow for precise control over the extent of albumin binding in different systems.

### 3.3 Nanocapsule Modification (Size Reduction)

**Figure 5:**
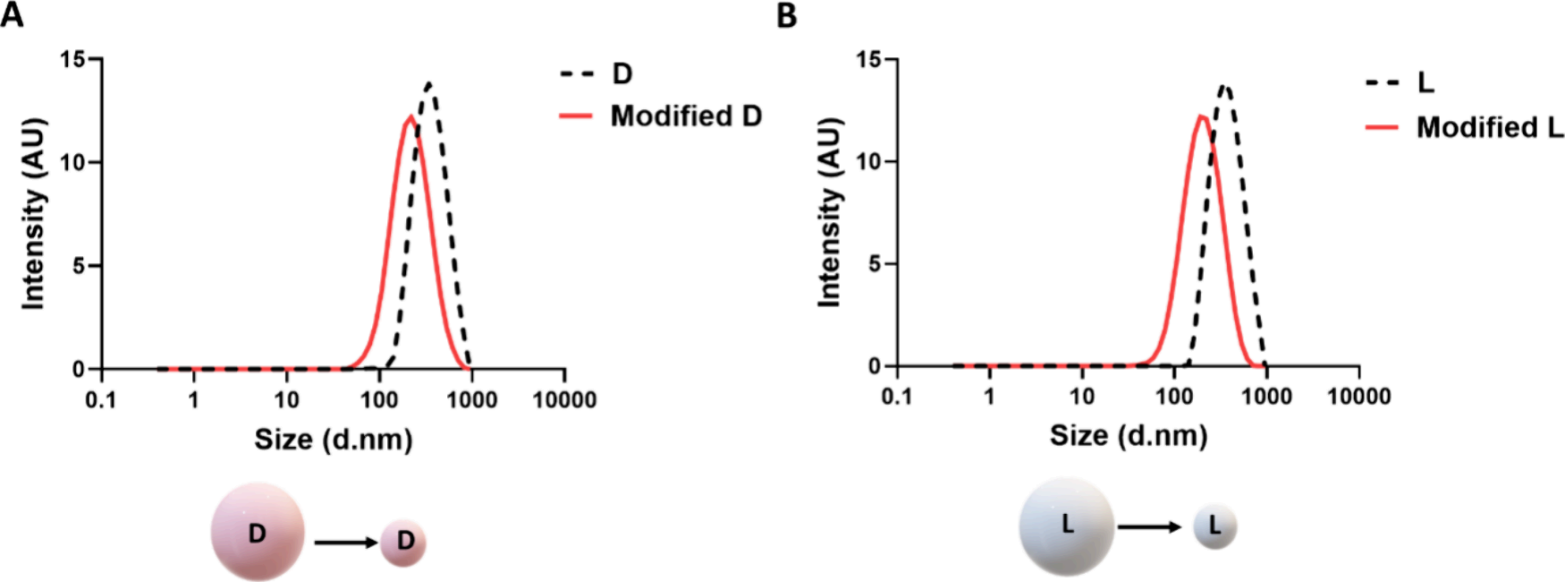
Representative size distributions by intensity of chiral polyurea nanocapsules before (dashed line) and after (solid line) size modification of (A) d-nanocapsules and (B) L- nanocapsules.

Because the ideal average particle size for systemic administration is typically smaller than 400 nm, in the range of ∼50–250 nm, size reduction was necessary in order to achieve a biorelevant nanoparticular system.^27^ We have significantly minimized the capsule size, rendering it suitable for systemic administration. This was done by empirically modifying the reaction conditions. We uncovered that elevating the polyaddition reaction temperature to 62 °C with tight temperature control and changing the stabilizing agent to hydrolyzed PVA, results in smaller nanocapsules. These capsules are further termed “modified nanocapsules” or “modified shells”. The size distributions of the modified shells were 184 ± 1 nm for d-nanocapsules and 204 ± 2 nm for l-nanocapsules, with polydispersity indexes of 0.29 ± 0.02 and 0.15 ± 0.03 for d- and l-nanocapsules, respectively. Size reduction for both chiral configurations is demonstrated in Figure 5.

**Figure 6:**
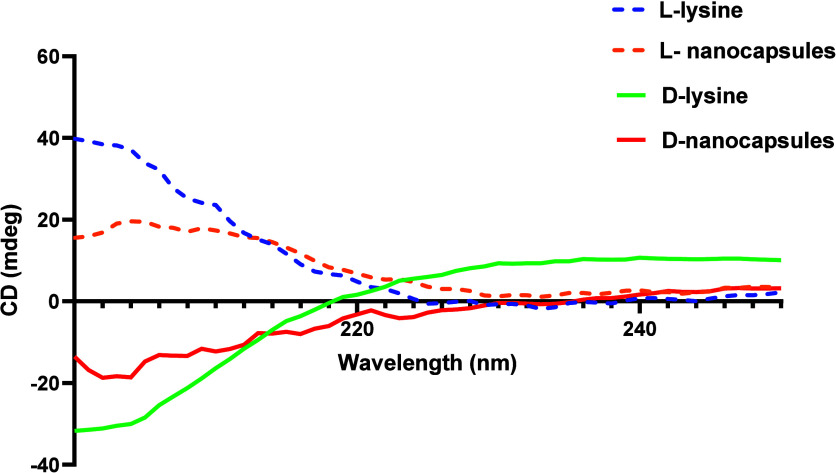
CD spectra of modified d- and l-nanocapsules. The spectra are compared to those of free amino acid enantiomers. The spectra of the nanocapsules show strong opposite signals that correspond well with the spectra of free lysine amino acid enantiomers, which confirm the chirality of the capsules.

All modified shells did not bear any surface charge, as confirmed by their almost neutral ζ-potentials. The ζ-potential values of modified shells were −2.7 ± 0.4 and −2.0 ± 0.2 mV for d-nanocapsules and l-nanocapsules, respectively. Their chirality was evaluated against those of their corresponding chiral amino acid monomers. Figure 6 shows CD spectra with strong negative and positive signals for d- and l-modified nanocapsules, respectively, which correlate well with their corresponding free amino acid enantiomer spectra.

**Figure 7:**
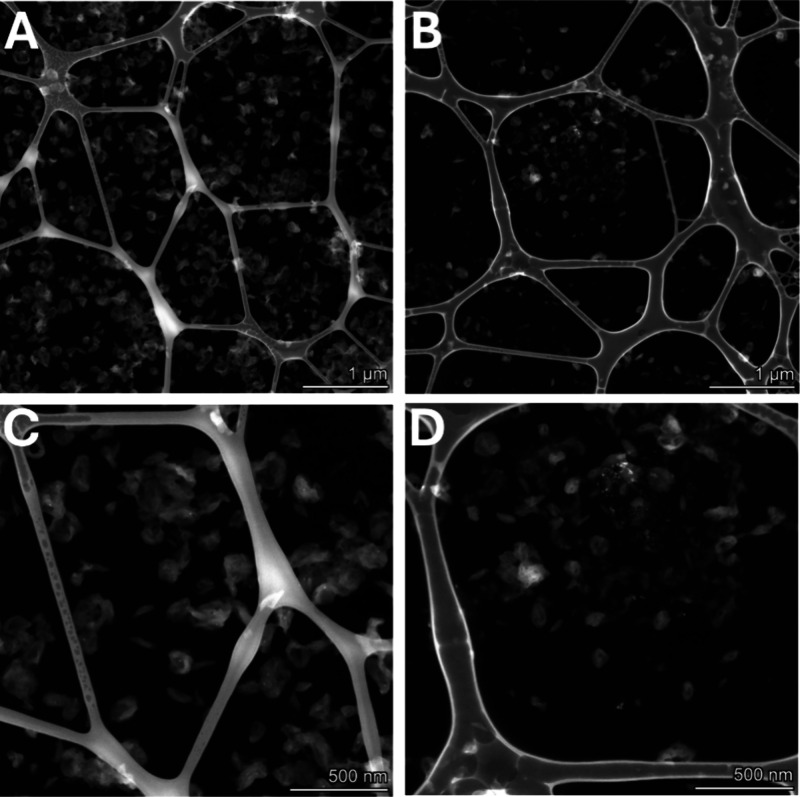
STEM images of (A) modified d-nanocapsules and (B) modified l-nanocapusles. Zoomed-in images of (C) modified d-nanocapsules and (D) modified l-nanocapsules.

The size and morphology of the modified chiral nanocapsules were then characterized by using scanning transmission electron microscopy (STEM). For both chiral configurations, sizes recorded by STEM correlated well with the DLS results for the modified capsules (Figure 7). It can be seen in these images and throughout all acquired imaging data that d-nanocapsules have predominantly donut shape morphology with a strong central depression, while the l-nanocapsule shape is closer to a folded sphere and has a much less pronounced central depression. Although experimental conditions during microscopy imaging can affect particle shape, the use of the same imaging protocol for both chiral configurations suggests an intrinsic shape difference. The prevalences of donut-shaped particles with a central depression effect were calculated for d- and l-nanocapsules and were found to be 81 and 8%, respectively.

The modified capsules exhibited good size reproducibility and were stable in storage in 0.4 w/v % aqueous dispersions for at least 6 months (probably owing to a steric stabilization of polymeric shells). The sizes measured after a 6-month storage period were 186 ± 2 nm for d-nanocapsules and 203 ± 2 nm for l-nanocapsules, with polydispersity indexes of 0.32 ± 0.01 and 0.21 ± 0.02 for d- and l-nanocapsules, respectively.

### 3.4 Albumin Adsorption onto Modified Chiral Polyurea Nanocapsules

**Figure 8:**
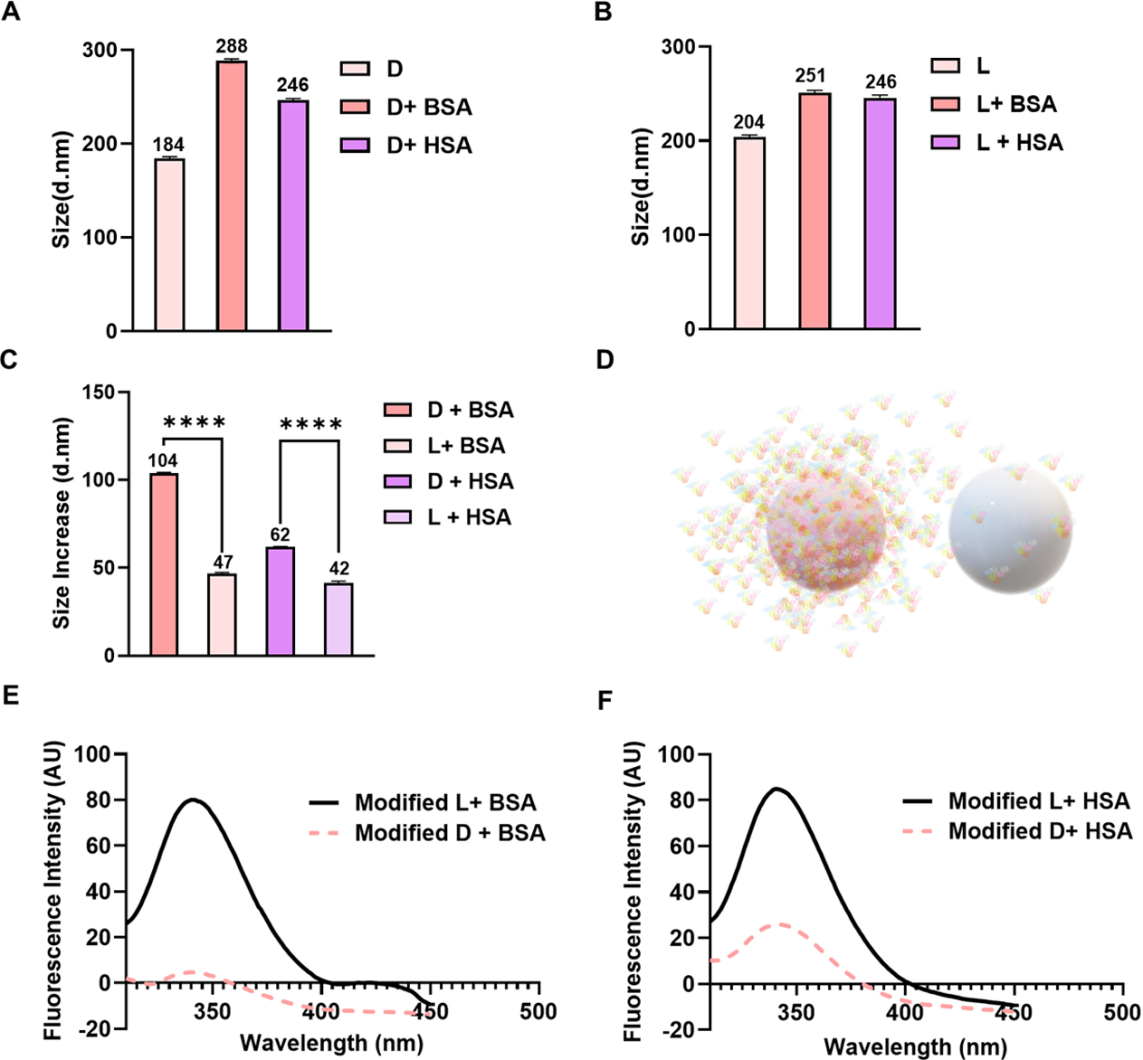
Interactions of modified chiral polyurea nanocapsules with albumins. (A) Representative size of modified d-polyurea nanocapsules before and after the adsorption of BSA and HSA. (B) Representative size of modified l-polyurea nanocapsules before and after adsorption of BSA and HSA. (C) Comparative analysis of the size increase after protein adsorption on d- and l-polyurea nanocapsules. Statistical analysis was performed by one-way analysis of variance with all pairwise multiple comparison procedures done by Tukey’s test. Symbols: ****P ≤ 0.0001. (D) Graphic presentation of protein adsorption on chiral capsules. (E) Fluorescence spectra after BSA equilibration with d-nanocapsules (dashed spectrum) and with l-nanocapsules (solid spectrum). (F) Fluorescence spectra after HSA equilibration with d-nanocapsules (dashed spectrum), and with l-nanocapsules (solid spectrum).

We employed albumins from different sources to investigate their possible interactions with the modified nanocapsules. First, possible interactions with BSA were evaluated. Next, to estimate the relevance to human therapy, the interactions with human serum albumin (HSA) were assessed. Size, albumin fluorescence quenching, and ζ-potential values were measured after the equilibration of modified capsules with the albumins. In addition, we calculated the concentrations of free albumin in the dispersion before and after incubation with the nanocapsules. Figure 8 and Figure S3 demonstrate differential size increases and fluorescence quenching after modified nanocapsule interactions with the albumins.

After interactions with BSA, the size of modified capsules increased from 184 ± 2 nm to 288 ± 2 nm for d-nanocapsules and from 204 ± 2 to 251 ± 3 nm for l-nanocapsules (Figure 8 and Figure S3). ζ-potential increased in its absolute value from −2.7 ± 0.4 mV to −22.3 ± 2.6 mV for d-nanocapsules and from −2.0 ± 0.2 mV to −11.2 ± 2.3 mV for l-nanocapsules. Modified d-nanocapsules quenched the BSA fluorescence stronger than modified l-nanocapsules (Figure 8E). By evaluating the free protein concentration, we estimated that the concentration decreased from 0.1% to 0.026% after the equilibration of BSA with modified d-nanocapsules and from 0.1% to 0.071% after its equilibration with modified l-nanocapsules, indicating that the amount of protein that was adsorbed to d-capsules was approximately 2.6 times greater.

Size of modified capsules following equilibration with HSA increased from 184 ± 2 nm to 246 ± 2 nm for d-nanocapsules and from 204 ± 2 to 246 ± 3 nm for l-nanocapsules (Figure 8 and Figure S3). The ζ-potential increased in its absolute value from −2.7 ± 0.4 to −20 ± 0.8 mV for d-nanocapsules and from −2.0 ± 0.2 to −13.4 ± 1.8 mV for l-nanocapsules after the interactions with HSA.

d-nanocapsules quenched the HSA fluorescence stronger than l-nanocapsules (Figure 8F). The free protein concentration decreased from 0.1% to 0.038% after the equilibration with modified d-nanocapsules, and from 0.1% to 0.071% after equilibration with modified l-nanocapsules indicating the amount of protein that was adsorbed to d-nanocapsules was approximately 2.1 times greater compared with their l-counterparts.

Thus, we observed that these smaller and more biorelevant chiral polyurea nanocapsules exhibit significant stereoselective interactions with both BSA and HSA. This was confirmed by (1) a larger size increase of d-nanocapsules compared to their l-counterparts in all experiments; (2) significantly more pronounced tryptophan residue fluorescence quenching of proteins observed with d-nanocapsules in all experiments; (3) a stronger negative ζ-potential measured on d-nanocapsule surface after equilibration with albumins.

The greater size increase of d-nanocapsules compared with l-nanocapsules can be explained by the formation of harder and denser protein corona due to the preferential adsorption of protein to their surfaces.^28^ This dense corona also accounts for the more negative ζ-potential stemming from the tighter decoration of nanocapsule surface with albumins that have an isoelectric point around 5 and, therefore, are negatively charged at pH = 7.4 of PBS medium.^29^

Significantly more pronounced protein tryptophan residue (Trp) fluorescence quenching observed with d-nanocapsules compared with their l-counterparts demonstrates a stronger association of albumin with d-nanocapsules. Trp fluorescence quenching evaluation is commonly used to determine the strength and mechanism of albumin interactions. The fluorescence of albumins is controlled by all of the fluorescent amino acids in their structure. Thus, HSA has 18 fluorescent tyrosine residues (Tyr), 31 weakly fluorescent phenylalanine residues (Phe), and one strongly fluorescent tryptophan residue (Trp-214) located in a hydrophobic pocket. BSA comprises 20 Tyr, 27 Phe, and two Trp residues: Trp-213, analogous to HSA Trp-214, and more superficial Trp-134.^30^ It is widely recognized that irradiating albumins at 280 nm results in the excitation of both Tyr and Trp residues, whereas 295 nm irradiation results in the selective excitation of Trp.^30^ Because we excited the samples at 295 nm, we mostly expected to see Trp fluorescence. Trp fluorescence is highly sensitive to the polarity of the surrounding environment; therefore, when albumins are adsorbed to a less polar surface, fluorescence shifts in its emission spectrum to lower wavelengths and fluorescence quenching of the sample is observed. It is evident that in the case of BSA, the shift is stronger due to the presence of an additional Trp residue, Trp-134.

### 3.5 Surface Morphology and Elemental Composition Studies

**Figure 9:**
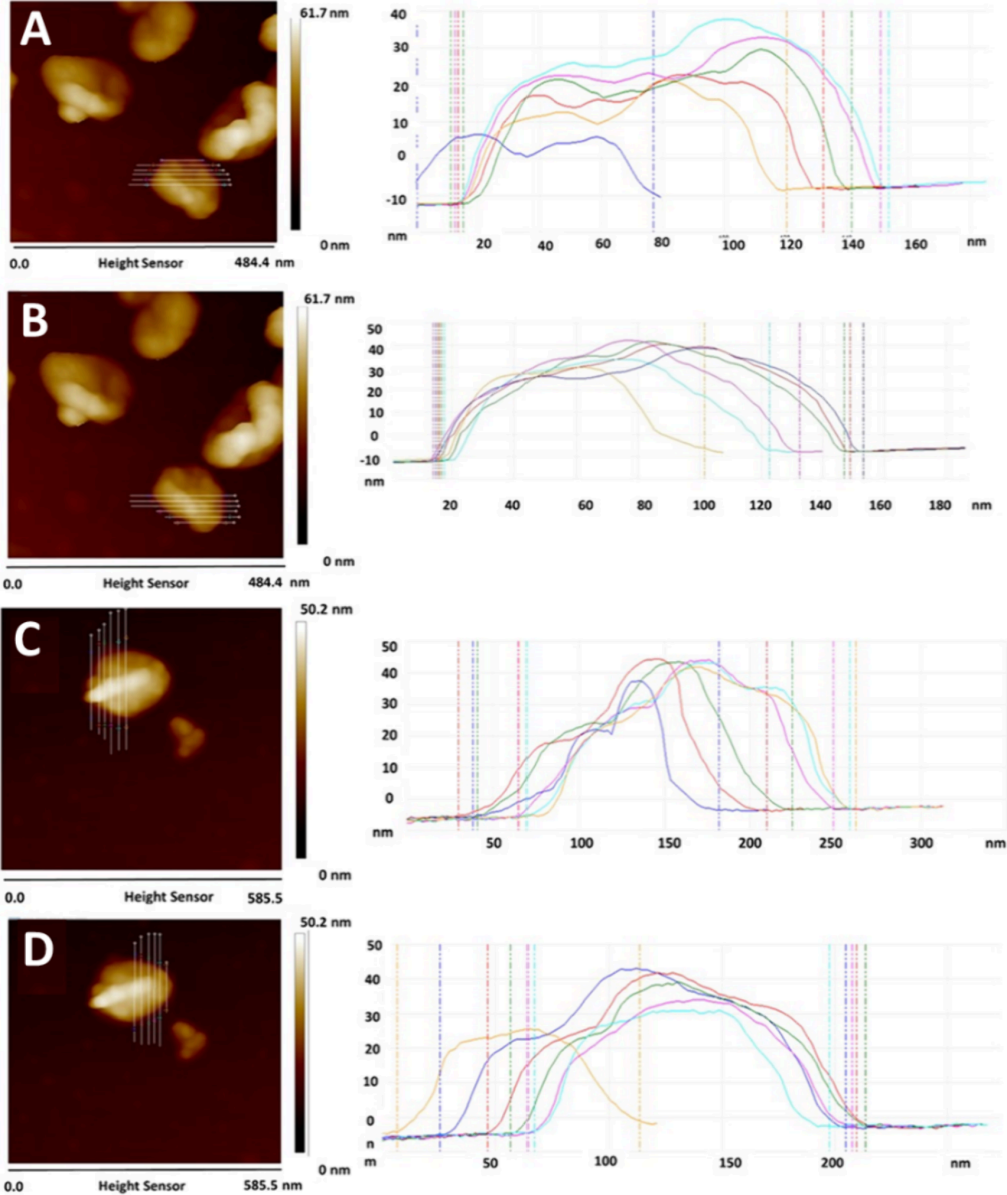
AFM images of d-capsules (A, B) and l-capsules (C, D). Cross-sectional profiles along the d-nanocapsules, and the l-nanocapsules marked with white lines in different regions.

To evaluate whether the extent of protein adsorption onto the nanocapsule surface can be impacted by the differences in the shell surface morphology, the surface topography of the modified d- and l-nanocapsules was measured with an AFM. Figure 9 and Figures S4 and S5 provide an in-depth analysis of different cross-section profiles for capsules of both chiral configurations.

**Figure 10:**
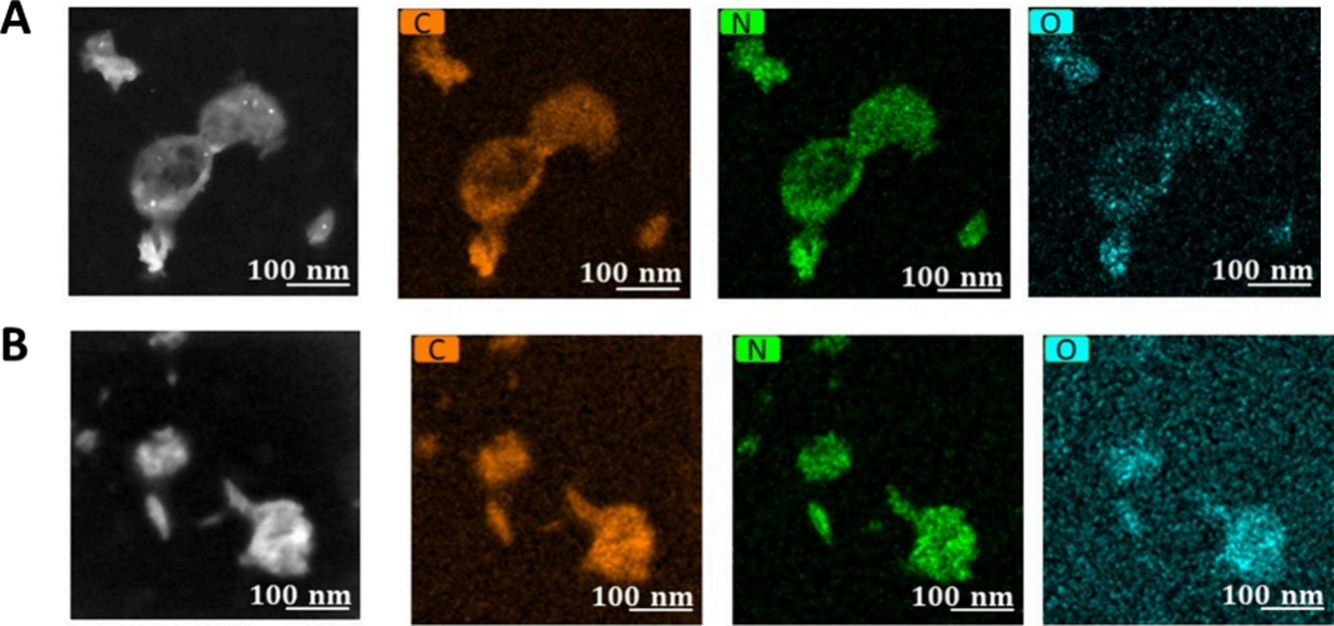
Elemental mapping (carbon, nitrogen, and oxygen) of polyurea nanocapsules using EDS of (A) d-polyurea nanocapsules and (B) l-nanocapsules. STEM images can be seen on the left image of each panel.

It is evident that surface morphology profiles of d-nanocapsules correspond well with the presence of the central depression (at least for some of the cross sections), while this phenomenon was not observed for l-nanocapsules (Figure 10, Figures S4 and S5). We considered the possibility that the measured l-capsule position simply hinders the central depression; however, other capsules that we analyzed demonstrate a similar trend (Figure S4).

We further evaluated the elemental composition of the nanocapsules using energy depressive X-ray spectroscopy (EDS, Figure 10). Similar presence of carbon, nitrogen, and oxygen elements was detected on the shells of both chiralities, as expected.

Together with the detailed analysis of the capsule shape in STEM images, these findings showcase the difference between the shape and surface morphology of d- and l-nanocapsules, despite both chiral capsules being composed of polyurea. This information can shed important light on the mechanism of the stereoselective interactions of nanocapsules with albumins. The significantly higher affinity of albumins for d-nanocapsules described above is indeed quite puzzling. In the case of small molecules, stereoselective interactions with albumins can be explained by a better fit of one of the enantiomers into different domains on the 3D structure of the proteins, allowing for stronger hydrophobic or electrostatic interactions. However, this can hardly explain strong stereoselectivity in interactions with the capsules in the size range of ∼184–384 nm. The possible interaction mechanism of albumins with neutral polymeric shells can involve the formation of multiple hydrogen bonds between the polyurea backbone and albumins and hydrophobic interactions between the hydrophobic domains of albumins and the nanocapsules. Yet, those possible interactions do not necessarily explain stereoselectivity because the chemical nature and functional groups are similar in the nanocapsules of both chiral configurations, as confirmed by EDS.

On the other hand, there is the notion that microscopic chirality may control macroscopic morphology, resulting in exposure or hindrance of the functional groups important for interactions.^31,32^ This notion was further confirmed by STEM and AFM analyses. A thorough evaluation of capsule shape and surface morphology revealed that chiral polymerization can affect both of these parameters, indeed controlling the macroscopic morphology. This morphological difference can also explain a slight (∼10%) difference between the initial sizes of d- and l-nanocapsules and could stem from the fact that chiral polymerization affects both the surface and the curvature of the shell.

**Figure 11:**
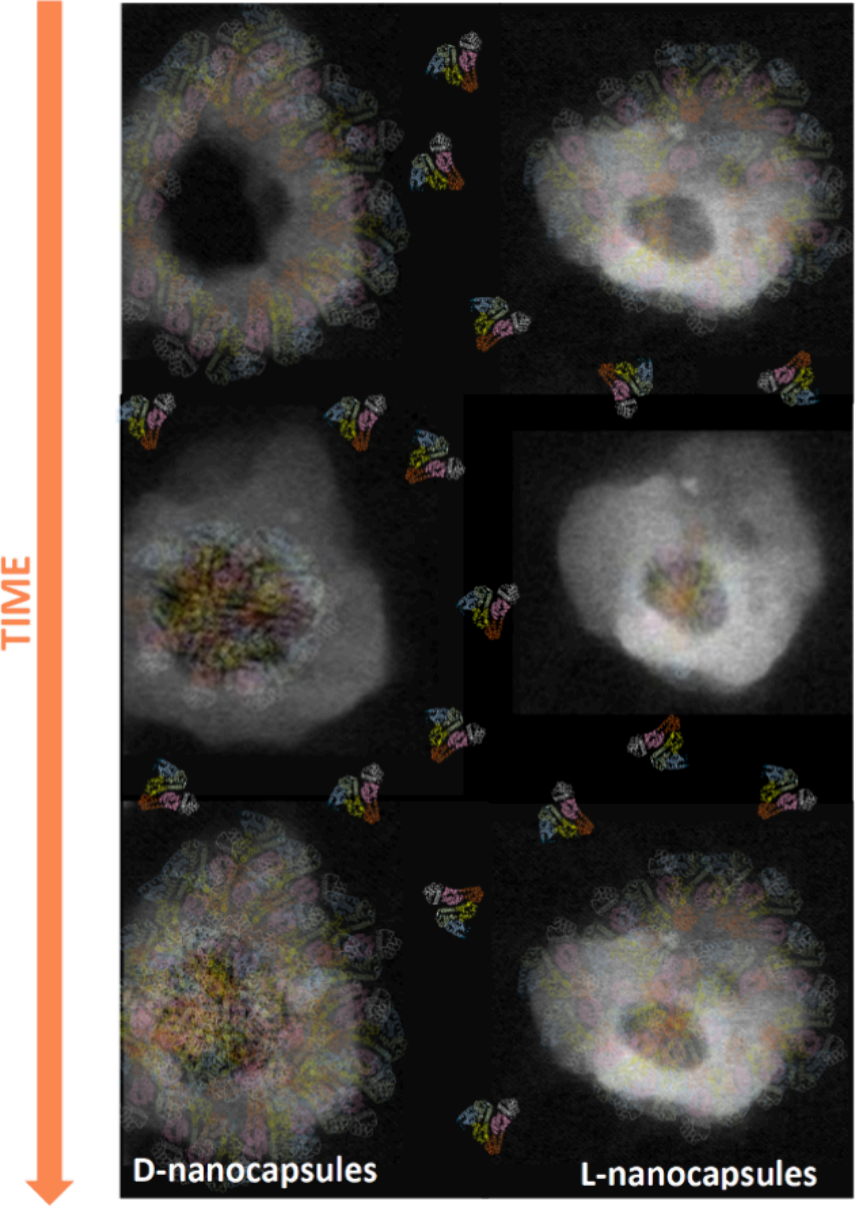
Simulation of dynamic albumin adsorption on the surface of chiral nanocapsules, with preferential adsorption on d-nanocapsules.

This may cause differences in the ease of adsorption and in the number of exposed functional groups that have an affinity for albumin.^33−35^ STEM images show a different final shape of the chiral nanocapsules. Whereas the majority of the l-nanocapsules have the typical capsule shape, many d-nanocapsules have a concave, donut-like shape, with a pronounced central depression effect. AFM cross-sectional profiles along the chiral nanocapsules also demonstrate that d-nanocapsules have an overall rougher surface with the central depression effect. This difference in shape and surface morphology may cause alterations in the ease of adsorption and in the number of exposed functional groups that have an affinity for albumin. Moreover, the central depression phenomenon may allow additional protein molecules to enter the formed cavity and maximize their interactions with the d-capsule surface (Figure 11).

### 3.6 Biocompatibility and Cellular Internalization of Chiral Polyurea Nanocapsules

**Figure 12:**
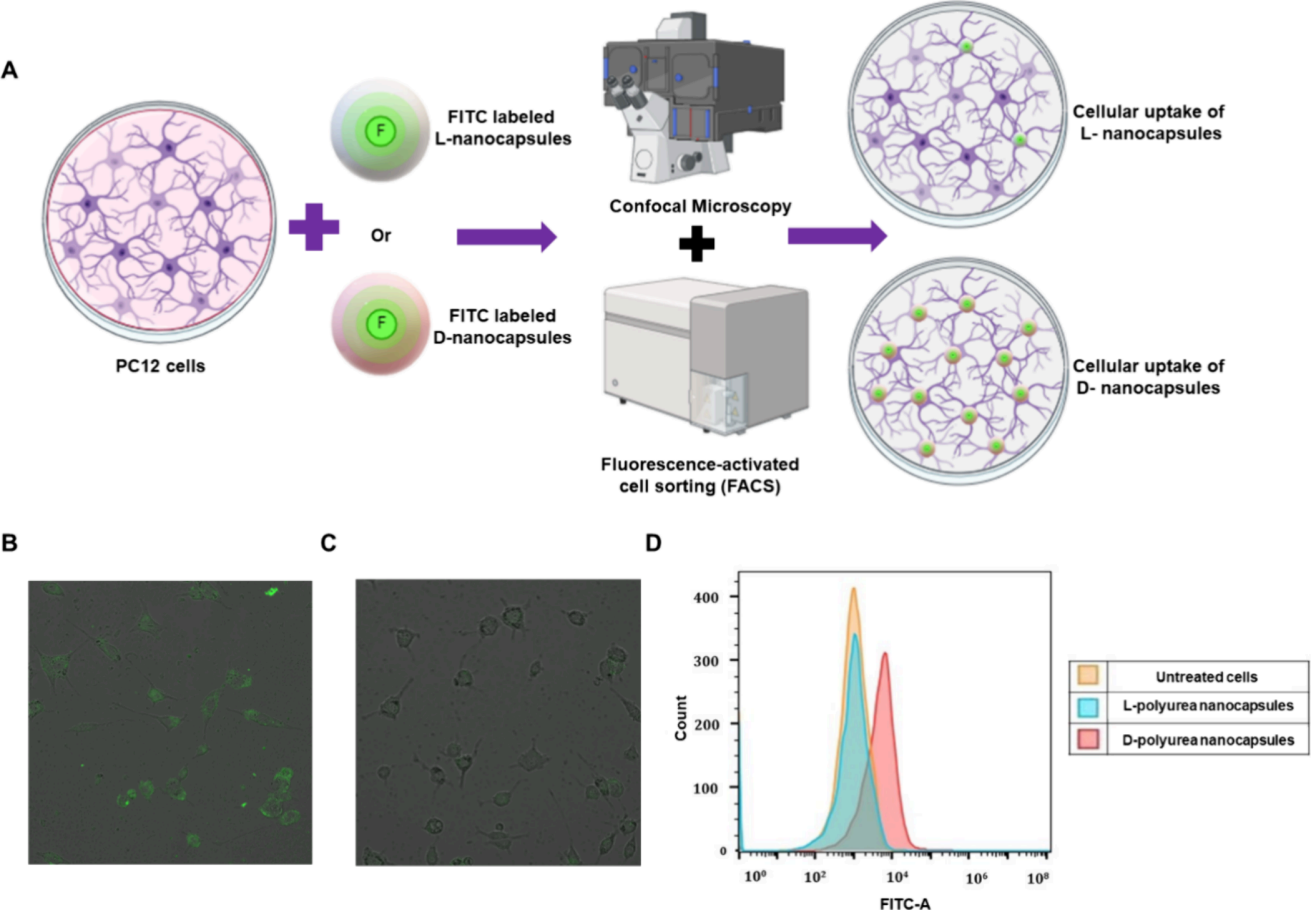
Cell internalization experiments. (A) Schematic representation of the experiments: FITC-loaded nanocapsules were incubated for two h with PC12 cells, and then the cells were imaged by confocal microscopy or sorted by FACS. (B) Confocal image of PC12 cells after 2 h incubation with 300 μg FITC-labeled-d-polyurea nanocapsules. (C) Confocal image of PC12 cells after 2 h incubation with 300 μg FITC-labeled-l-polyurea nanocapsules. (D) FACS analysis of FITC-labeled d- and l-polyurea nanocapsules cellular uptake into PC12 cells after 2 h of incubation

We next decided to test the biocompatibility of the nanocapsules and explore whether albumin adsorption by the nanocapsules can facilitate its internalization by cells. In the first step, we performed an MTT assay on PC12 cells, a commonly used cell line for evaluation of cellular effects of nanoparticle internalization.^36,37^ The cells were exposed to the modified nanocapsules of both chiralities for 48 h, and cell viability was not affected (Figure S6). Further, d- and l-modified nanocapsules were labeled with fluorescein isothiocyanate (FITC) and dispersed in the cell media of PC12 cells, which contains approximately 0.6% (w/v) of a fetal calf and horse albumins with a smaller percentage of other plasma proteins.^38^ The fluorescence intensities of the FITC-labeled d- and l-nanocapsules were similar (Figure S7). After 2 h of incubation, live cell imaging of PC12 cells was performed, and the results are shown in Figure 12B,C. FITC fluorescence intensities of cells incubated with d-nanocapsules and l-nanocapsules were 277.2 ± 65.3 and 107.7 ± 20.5 respectively. Based on the fluorescence signal, the cellular internalization of d-nanocapsules was approximately 2.5-fold greater than that of their l-counterparts. This is in good correspondence to the greater extent of albumin adsorption detected on d-nanocapsules with different albumins (∼2.6 and ∼2.1-fold preferential adsorption on d-nanocapsules with BSA and HSA respectively, as reported in Section 3.4).

In parallel, flow cytometry experiments (fluorescence-activated cell sorting, FACS) were conducted to detect the fluorescence intensity in the living cells, which directly correlates with the number of d- or l-polyurea nanocapsules internalized by the cells in each experiment. FACS results confirmed a superior ability of d-nanocapsules to permeate the cells, as shown in Figure 12D.

We infer that this significant difference in the cellular uptake of the nanocapsules is due to the higher adsorption of proteins from the cell medium. It is established that albumin coating significantly enhances cell internalization of nanoparticles.^8,11−14^ Multiple body fluids contain albumin, with blood plasma as its primary source. Hence, manipulating the chirality of nanocapsules that are expected to come into contact with these fluids can regulate their subsequent cellular uptake.

## 4 Conclusions

We have uncovered that chiral polyurea nanocapsules synthesized by polyaddition reaction between toluene 2,4-diisocyanate and d- or l-lysine enantiomers exhibit significant stereoselective interactions with albumins from different sources, despite their nearly neutral surface. These nanocapsules are biocompatible, nontoxic, stable in storage, and can be formed at a size range suitable for *in vivo* administration.

The interaction strength between the nanocapsules and albumin can be further controlled by incorporating varying amounts of chiral monomers into the synthesis reaction mixture. The interactions generally become more pronounced with the rise in the d-monomer content during the synthesis reaction. This ability to control the extent of albumin binding can play a pivotal role in drug development as it may enable control over the drug’s pharmacokinetics.

The significantly higher affinity of albumins to d-nanocapsules (2.1–2.6-fold) can be attributed to the nanocapsules’ chirality-dependent shape and surface morphology, as confirmed by STEM, AFM, and EDS analyses. d-nanocapsules exhibit a concave, donut-like shape with a pronounced central depression and a rougher surface, whereas l-nanocapsules are generally spherical with smoother surfaces. These morphological differences can affect albumin adsorption, likely due to variation in the number of exposed functional groups with an affinity for albumin.

Finally, substantially higher cellular internalization of d-nanocapsules was observed, probably due to the preferential adsorption of albumins and other proteins from the cell medium onto this configuration. Indeed, ∼2.5-fold greater internalization was recorded for d-nanocapsules based on fluorescence intensity, which corresponds well to 2.1–2.6-fold greater albumin adsorption recorded for this configuration.

## Supporting Information Available

Interactions of chiral nanocapsules with purified and plasma albumins, shells of mixed chirality, albumin adsorption onto modified chiral polyurea nanocapsules, surface morphology and elemental composition studies, effect of modified d- and l- nanocapsules on cell viability, and fluorescence intensity of chiral FITC-labeled polyurea nanocapsules (PDF)

## Abbreviations

AFM: atomic force microscopy
BSA: bovine serum albumin
DDW: double distilled water
DLS: dynamic light scattering
DMEM: Dulbecco’s modified Eagle’s medium
EDS: energy depressive X-ray spectroscopy
FACS: fluorescence-activated cell sorting
FITC: fluorescein isothiocyanate
HAADF: high-angle annular dark field
v/v: volume per volume
HSA: human serum albumin
PBS: phosphate buffered saline
PDI: polydispersity indexes
Phe: phenylalanine
PVA: poly(vinyl alcohol)
Rpm: rounds per minute
SD: Sprague–Dawley
STEM: scanning transmission electron microscopy
TDI: toluene 2,4-diisocyanate
Trp: tryptophan
Tyr: tyrosine
w/v: weight per volume
wt%: percentage by weight
