# Supplementary material for: Stereoselective Interactions of Chiral Polyurea Nanocapsules with Albumins
Source: ACS Appl Mater Interfaces. 2024 Aug 23;16(43):58168–79. doi: 10.1021/acsami.4c09565 (PMC11533163; doi:10.1021/acsami.4c09565)
Supplement: Supplementary file 1 — am4c09565_si_001.pdf [file am4c09565_si_001.pdf]

Supporting Information

Stereoselective Interactions of Chiral Polyurea Nanocapsules with Albumins

Amani Zoabi,[1] Adan Sultan,[1] Malak Abo Alhija, [1] Sergei Remennik, [2] Anna Radko,[2] Katherine Margulis[1**]

[1]The Institute for Drug Research, the School of Pharmacy, the Faculty of Medicine, The Center for Nanoscience and Nanotechnology, The Hebrew University of Jerusalem, Jerusalem 9112192, Israel.

[2] The Unit for Nanoscopic Characterization, The Center for Nanoscience and Nanotechnology, The Hebrew University of Jerusalem, Jerusalem 91904, Israel.

**Correspondence to: katy.margulis@mail.huji.ac.il

Interactions of chiral nanocapsules with purified and plasma albumins

To examine the adsorption of albumins to the nanocapsule surface, nanocapsule size increase and the quenching of tryptophan residues fluorescence of albumins before and after the incubation with nanocapsules were evaluated (**Figures S1, 3**).

It can be clearly seen that markedly greater albumin adsorption occurred on D-nanocapsules compared to their L-counterparts.

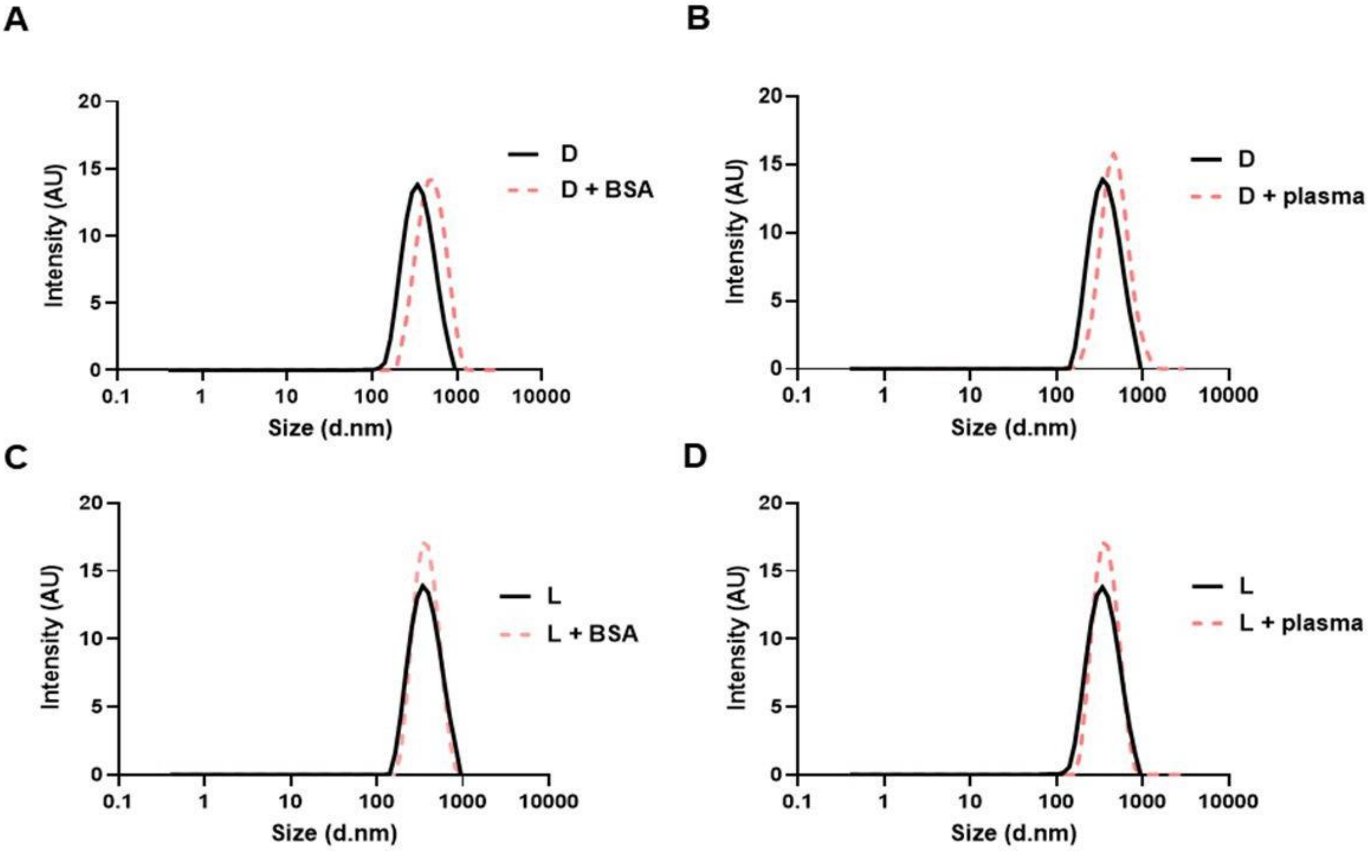

Figure 1. Interactions of chiral polyurea nanocapsules with albumins. **(A)** Representative size distribution by intensity of D-polyurea nanocapsules before (solid graph) and after (dashed graph) incubation with BSA. **(B)** Representative size distribution by intensity of D-polyurea nanocapsules before (solid graph) and after (dashed graph) incubation with plasma. **(C)** Representative size distribution by intensity of L-polyurea nanocapsules before (solid graph) and after (dashed graph) incubation with BSA. **(D)** Representative size distribution by intensity of L-polyurea nanocapsules before (solid graph) and after (dashed graph) incubation with plasma.

Shells of mixed chirality

We assumed that it would be possible to affect the extent of albumin adsorption onto the nanocapsules by introducing both enantiomeric monomers at different ratios during chiral shell synthesis. The increase in size of synthesized nanocapsules with mixed chirality before and after BSA adsorption is demonstrated in **Figures S2** and **4**.

It can be seen that the size increase becomes greater with the rise in the content of D-monomer during the synthesis reaction, except for the 50:50 ratio, which probably results in racemic capsules. We infer that the interactions between the nanocapsules and albumin become more pronounced with the increase in D-chirality on the nanocapsule surface.

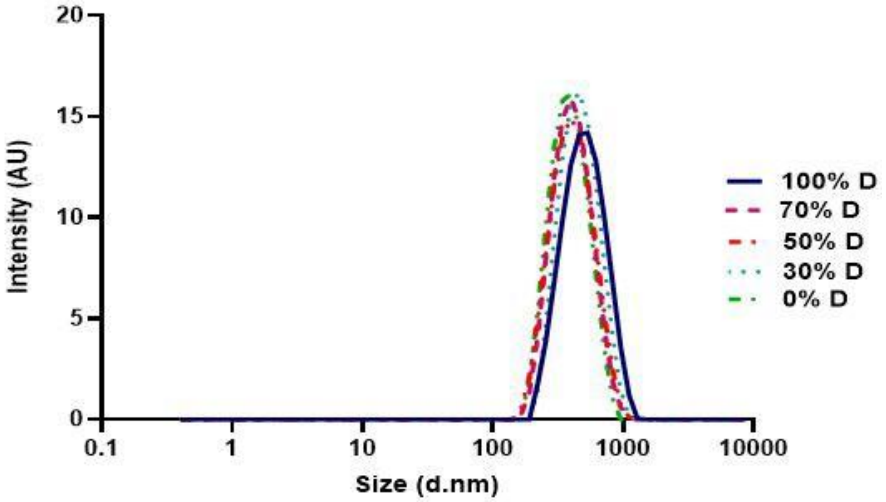

Figure S2. Representative size distributions by the intensity of chiral nanocapsules formed by different ratios between D- and L-lysine enantiomers after BSA adsorption.

Albumin adsorption onto modified chiral polyurea nanocapsules

We employed albumins from different sources, specifically bovine and human to investigate their possible interactions with modified (smaller) nanocapsules (Figurea S3, 8).

The results show significantly greater adsorption of the albumins on D-modified nanocapsules compared to their L-counterparts.

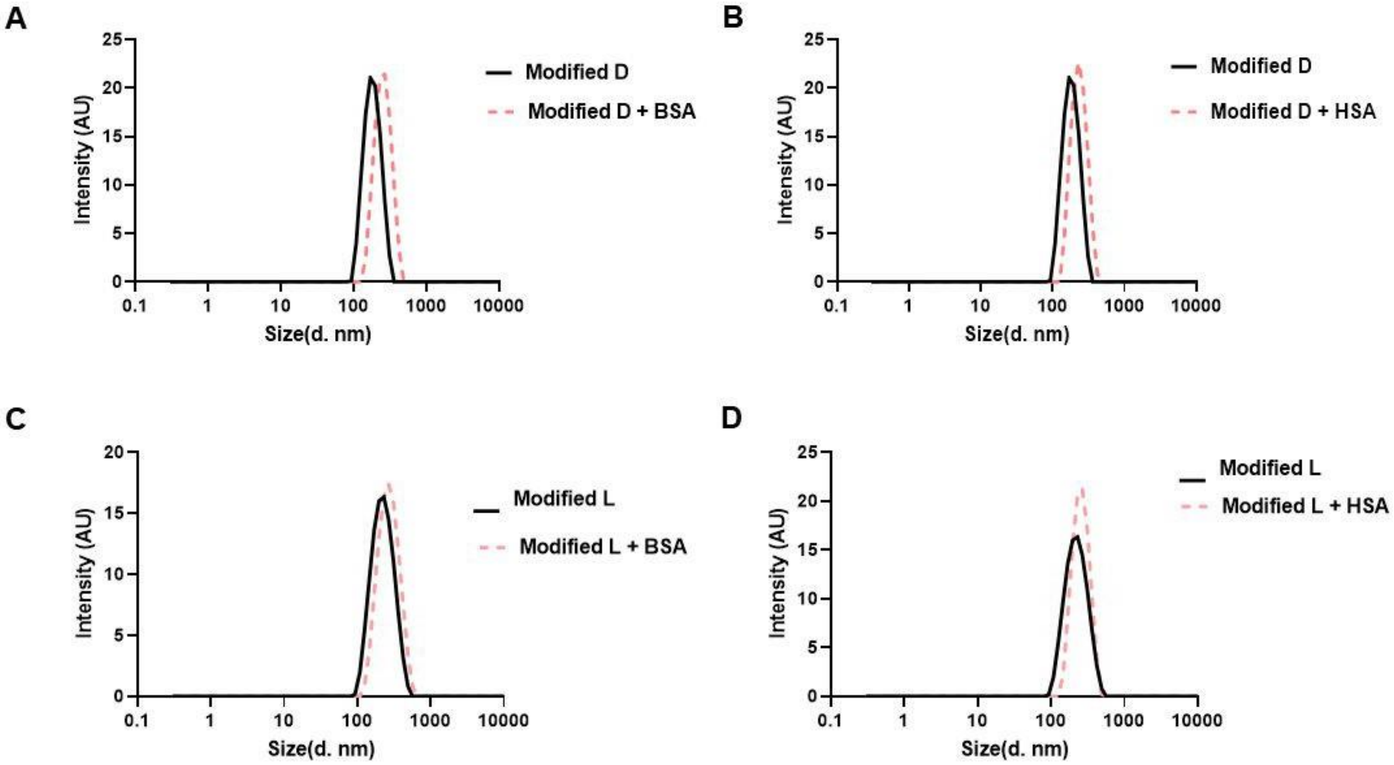

Figure S3. (A) Representative size distribution by intensity of D-polyurea nanocapsules before (solid graph) and after (dashed graph) incubation with BSA. **(B)** Representative size distribution by intensity of D-polyurea nanocapsules before (solid graph) and after (dashed graph) incubation with HSA. **(C)** Representative size distribution by intensity of L-polyurea nanocapsules before (solid graph) and after (dashed graph) incubation with BSA. **(D)** Representative size distribution by intensity of L-polyurea nanocapsules before (solid graph) and after (dashed graph) incubation with HSA.

Surface morphology and elemental composition studies

To evaluate whether the extent of protein absorption onto the nanocapsule surface can be impacted by the differences in the shell surface morphology, the surface topography of the chiral D- and L-nanocapsules was measured with an AFM. An in-depth analysis of multiple cross-section profiles of different capsules is shown in **Figure S4** (for L-nanocapsules), in **Figure S5** (for D-nanocapsules), and **Figure 9** for the nanocapsules of both chiralities.

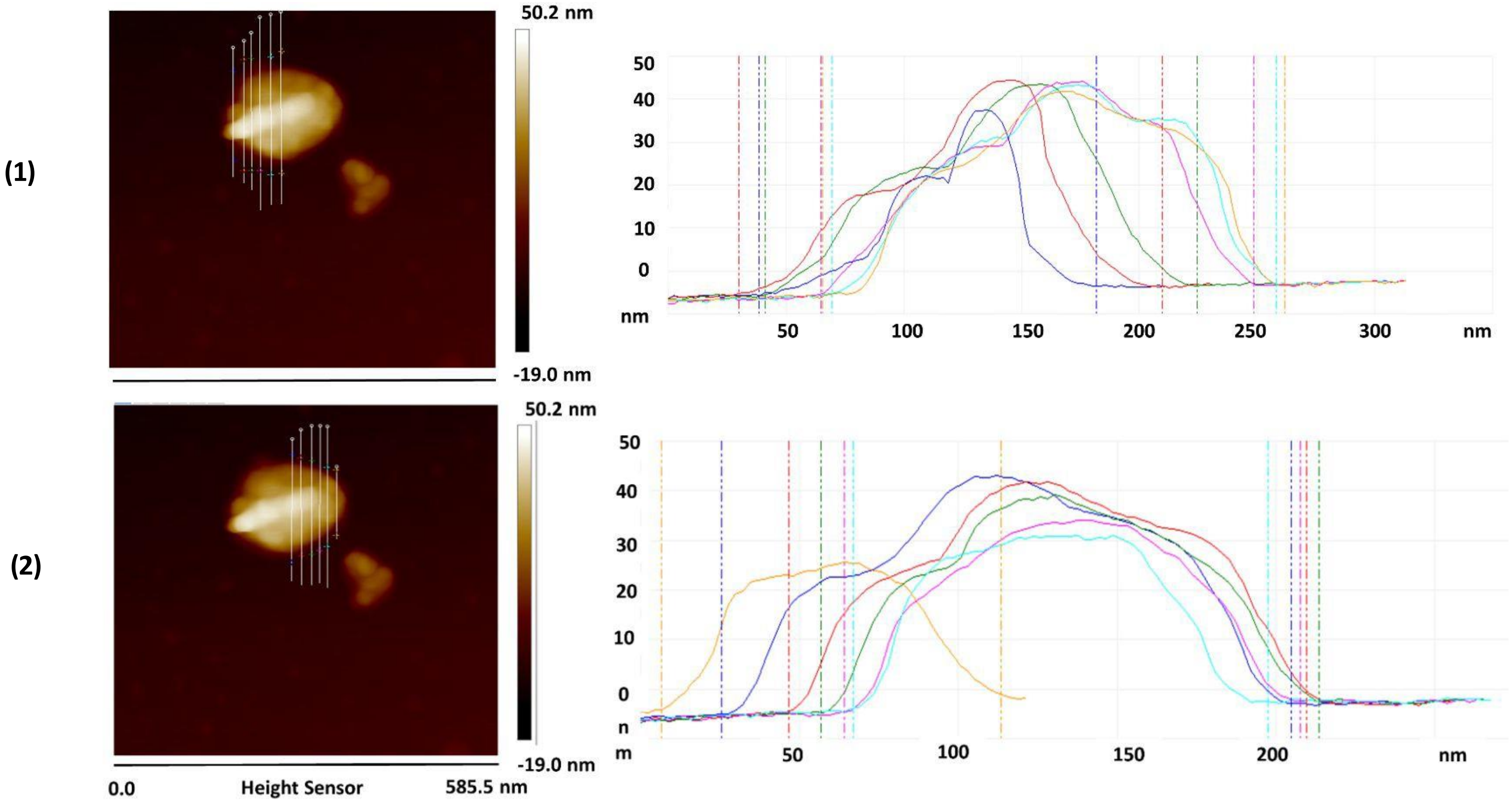

(3)

(4)

(5)

(6)

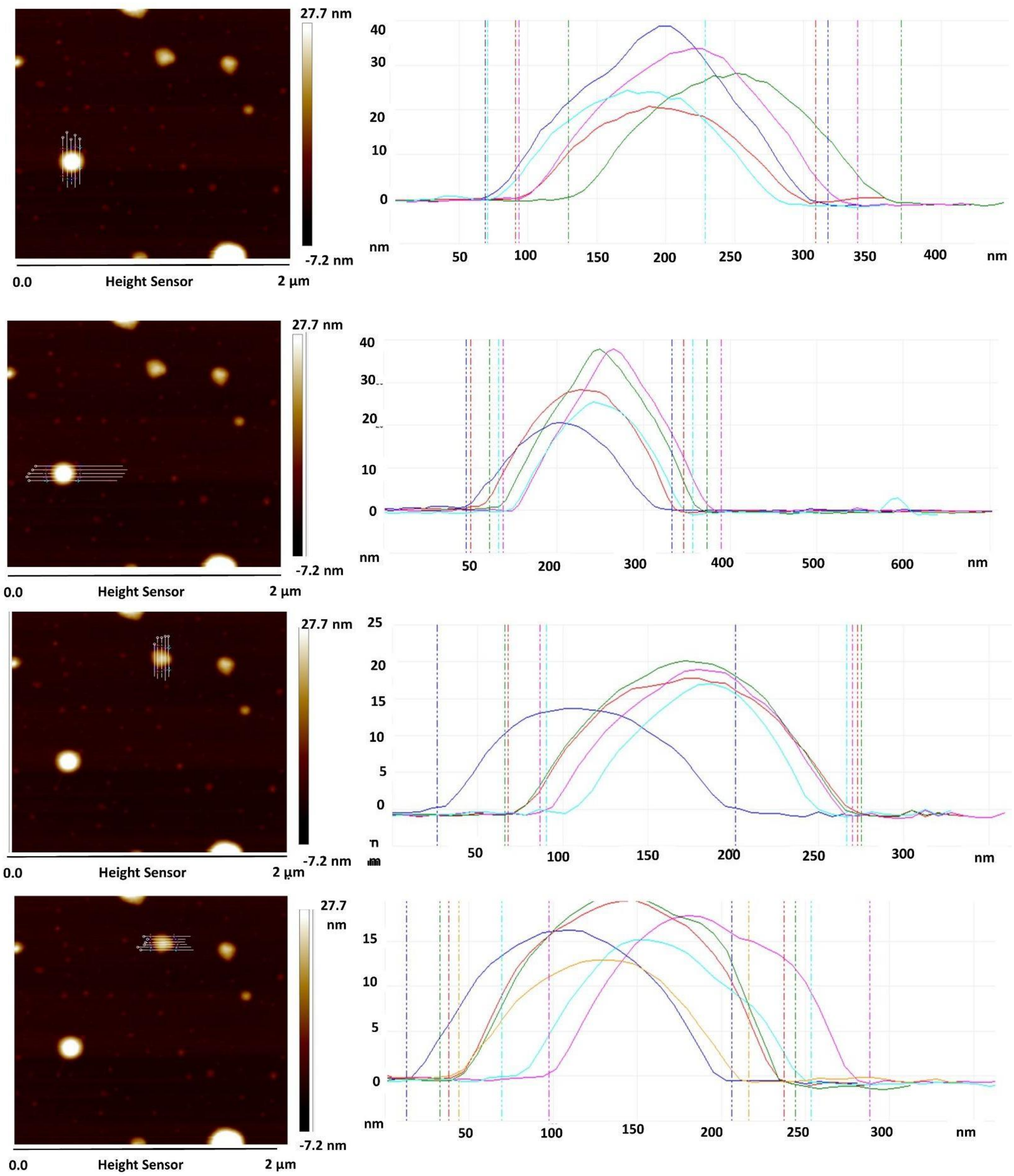

Figure S4. (1-6) Cross-section profiles of two different L-nanocapsules. Measured cross-sections are marked in white on the left panels, and every curve on the right panels represents a different cross-section profile.

(1)

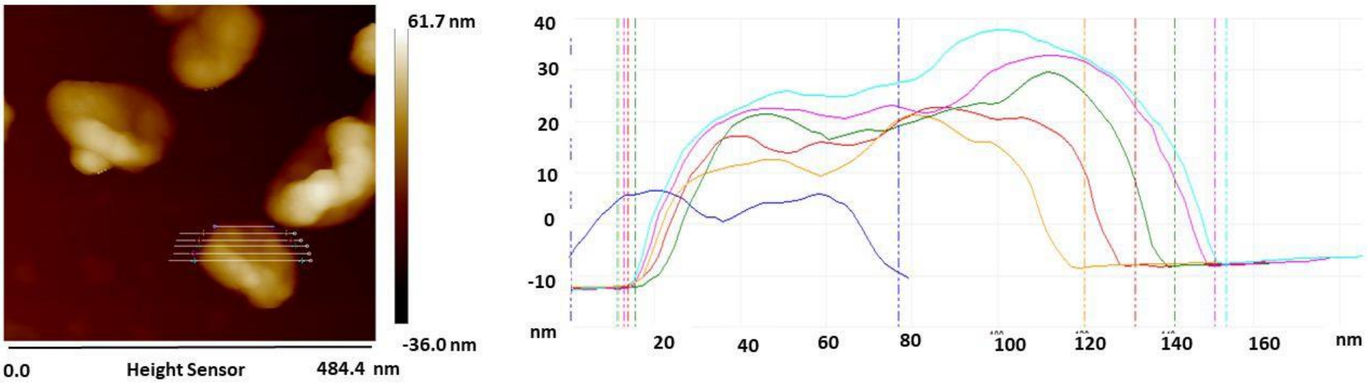
61.7 nm
-36.0 nm
0.0
Height Sensor
484.4 nm
40
30
20
10
0
-10
nm
20
40
60
80
100
120
140
160
nm

(2)

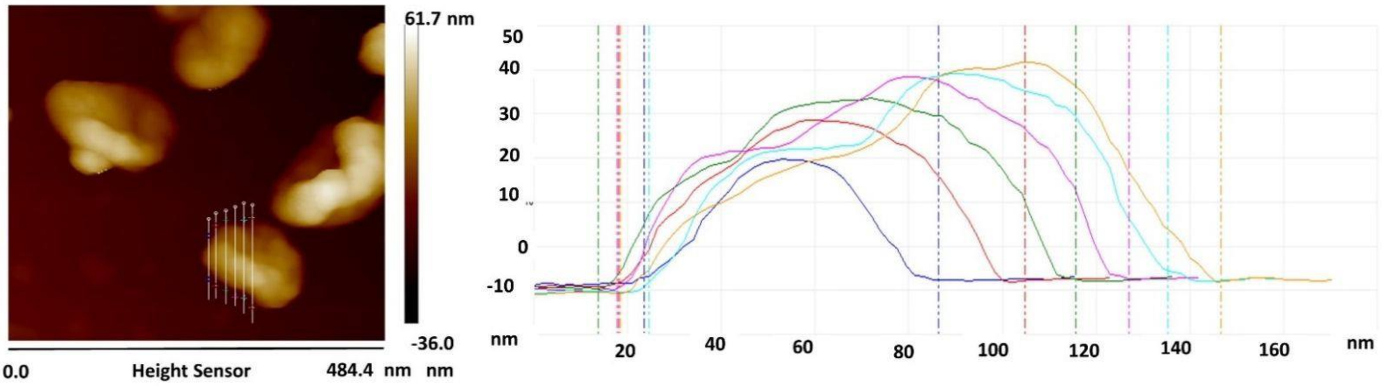
61.7 nm
-36.0
0.0
Height Sensor
484.4 nm nm
50
40
30
20
10
0
-10
nm
20
40
60
80
100
120
140
160
nm

(3)

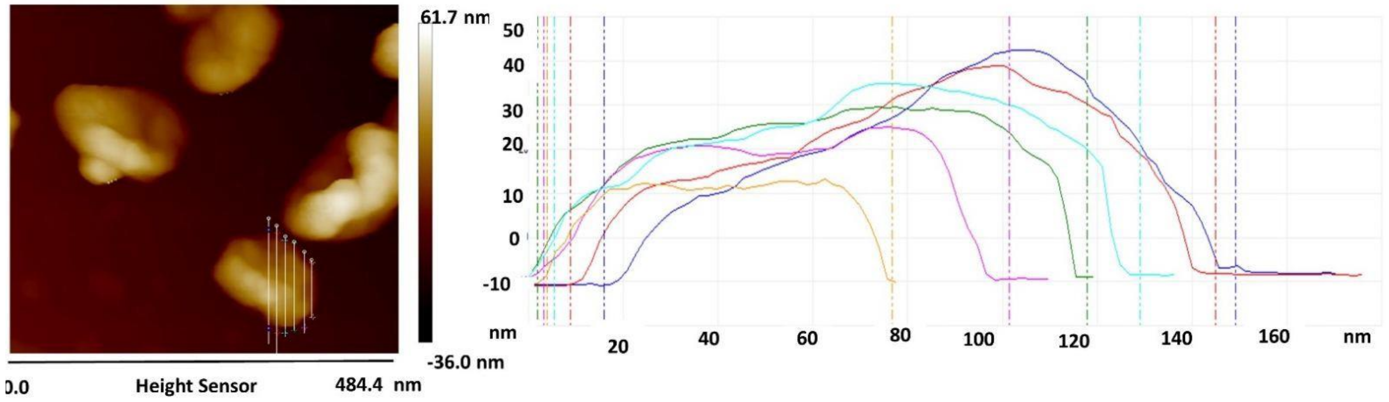
61.7 nm
-36.0 nm
0.0
Height Sensor
484.4 nm
50
40
30
20
10
0
-10
nm
20
40
60
80
100
120
140
160
nm

(4)

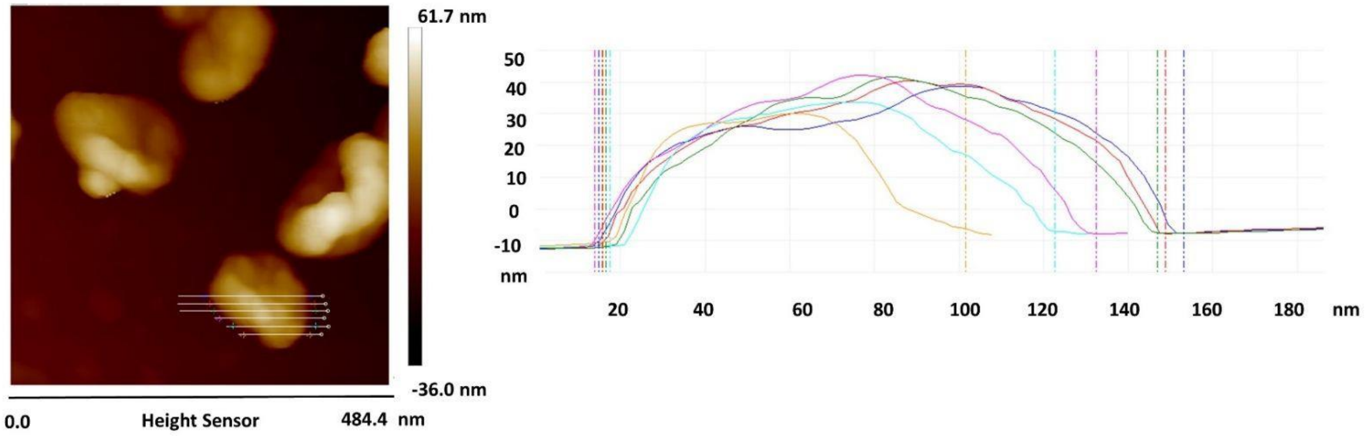
61.7 nm
-36.0 nm
0.0
Height Sensor
484.4 nm
50
40
30
20
10
0
-10
nm
20
40
60
80
100
120
140
160
180
nm

(5)

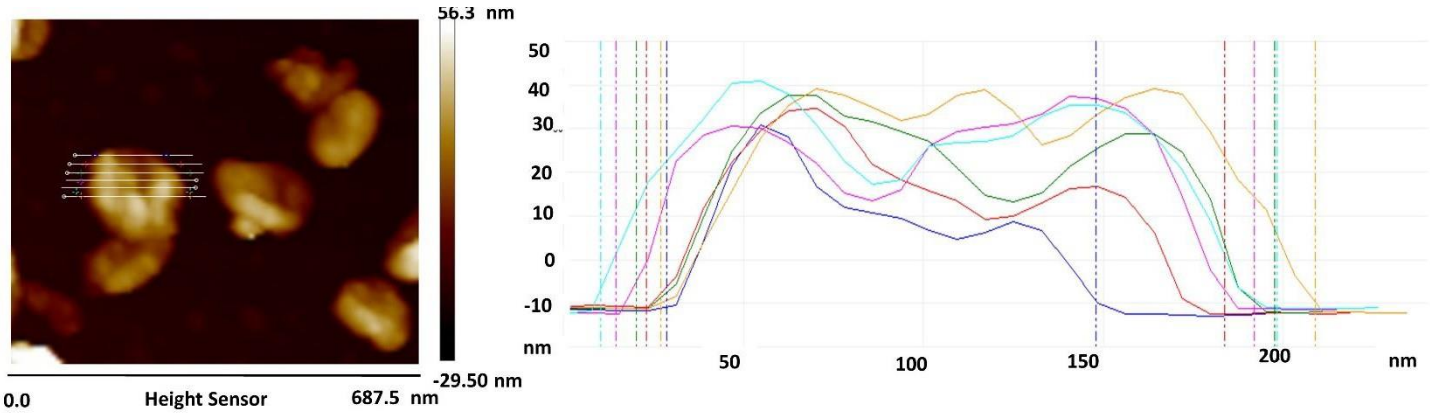
56.3 nm
-29.50 nm
0.0
Height Sensor
687.5 nm
50
40
30
20
10
0
-10
nm
50
100
150
200
nm

(6)

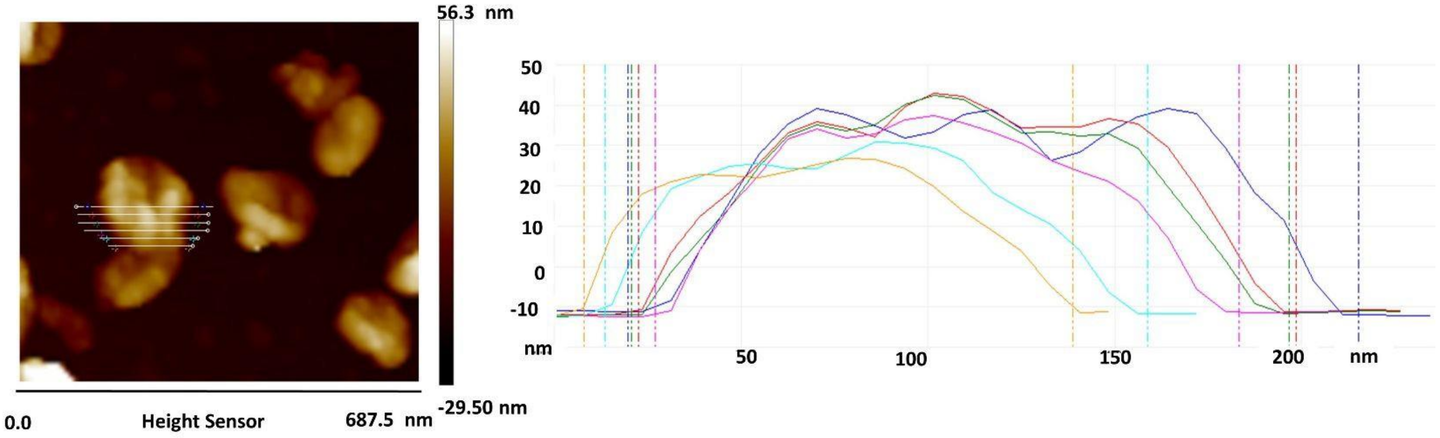
56.3 nm
-29.50 nm
0.0
Height Sensor
687.5 nm
50
40
30
20
10
0
-10
nm
50
100
150
200
nm

(7)

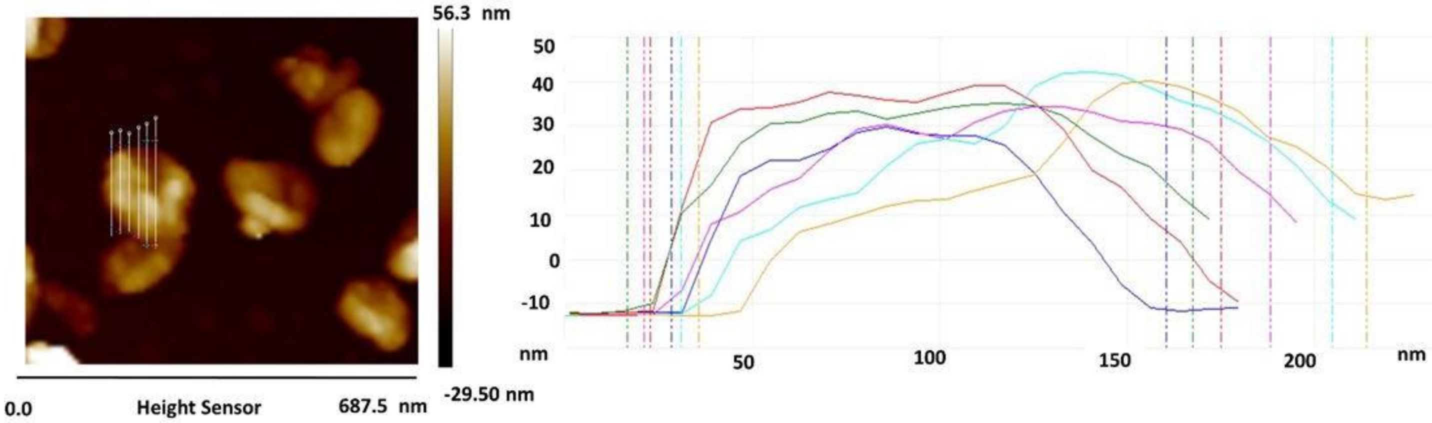
56.3 nm
-29.50 nm
0.0
Height Sensor
687.5 nm
50
40
30
20
10
0
-10
nm
50
100
150
200
nm

(8)

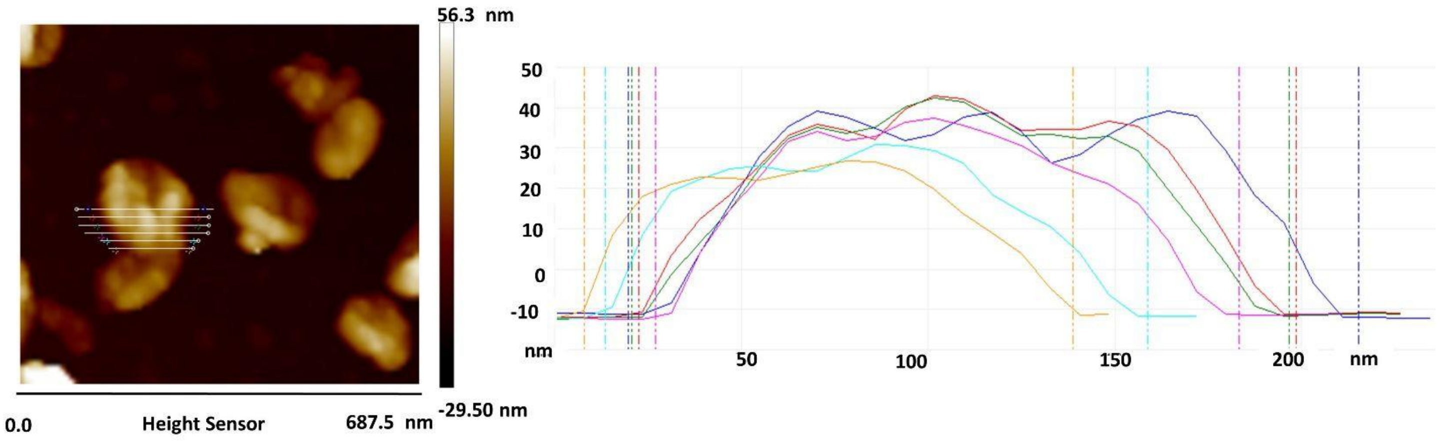
56.3 nm
-29.50 nm
0.0
Height Sensor
687.5 nm
50
40
30
20
10
0
-10
nm
50
100
150
200
nm

(9)

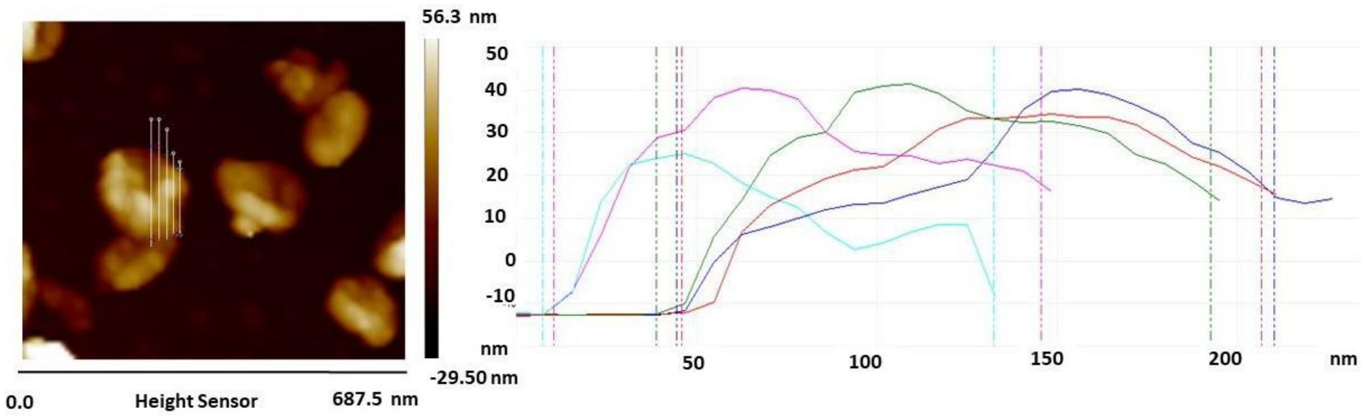

Figure S5. (1-9) Cross-section profiles of two different D-nanocapsules. Measured cross-sections are marked in white on the left panels, and every curve on the right panels represents a different cross-section profile.

It can be appreciated from **Figures 9, S4** and **S5** that the surface morphology profiles of D-nanocapsules show a central depression at least for some of the cross-sections, whereas this phenomenon is absent in L-nanocapsules.

These findings, along with a detailed analysis of the capsule shape in STEM images and elemental mapping collectively highlight the differences in shape and surface morphology between D- and L-nanocapsules.

Effect of modified D- and L- nanocapsules on cell viability

The effect of D- and L- polyurea nanocapsules on cell viability was assessed using an MTT assay. The cells were exposed to the nanocapsules for 48 hours at a 1mg/ml concentration dispersed in a cell culture medium without the utilization of organic solvents. Untreated cells were tested as a negative control. The data showed that both D- and L-polyurea nanocapsules did not affect the viability of cells over 48 hours **(Figure S6).**

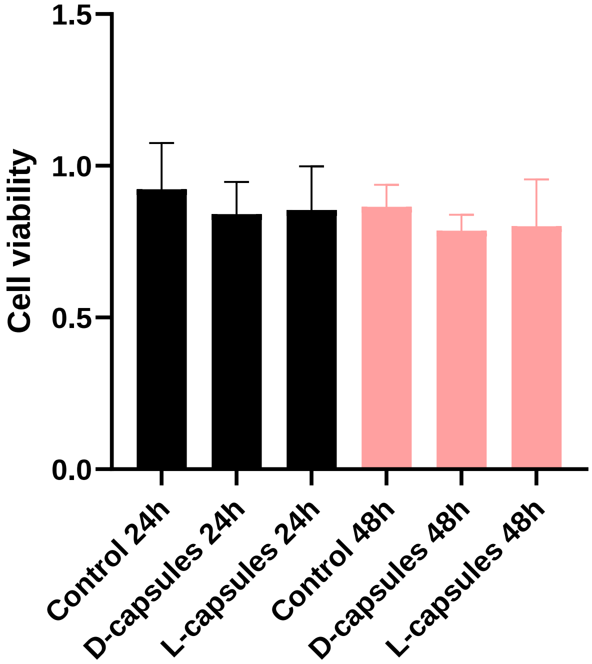

Figure S6. PC12 cell viability analysis using MTT assay. Control 24 h- the viability of untreated cells after 24 hours; D-capsules 24 h- the viability of cells treated with 1mg/ml D-polyurea nanocapsules after 24 hours; L-capsules 24 h- the viability of cells treated with 1mg/ml L-polyurea nanocapsules after 24 hours; Control 48 h- the viability of untreated cells after 48 hours; D-capsules 48 h- the viability of cells treated with 1mg/ml D-polyurea nanocapsules after 48 hours; L-capsules 48 h- the viability of cells treated with 1mg/ml L-polyurea nanocapsules after 48 hours. The data shown represents two separate experiments, and the values are given as mean ± SD. Statistical analysis was performed by one-way analysis of variance with all pairwise multiple comparison procedures done by Tukey test.

Fluorescence intensity of chiral FITC-labeled polyurea nanocapsules

The fluorescence intensity of FITC was measured using a spectrophotometer to ensure that the labeled D- and L-nanocapsules had the same fluorescence intensity before incubating with cells. Upon excitation at 498 nm, the fluorescence emission spectra were recorded in the 450 to 600 nm wavelength range. Both D- and L-nanocapsules loaded with FITC showed similar fluorescence intensity, 714.7 for D-nanocapsules and 720.2 for L-nanocapsules **(Figure S7)**.

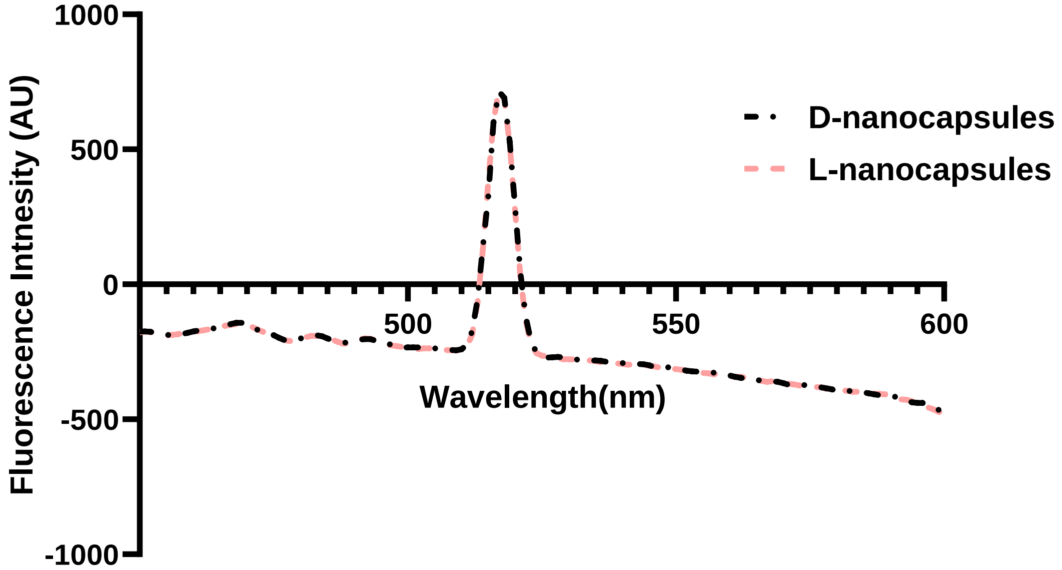

Figure S7. Fluorescence spectra of FITC-loaded D-nanocapsules (black spectrum) and FITC-loaded L-nanocapsules (pink spectrum).
